# Mapping cumulative compound hydrometeorological and marine-induced risks on the NW Mediterranean coast

**DOI:** 10.1038/s41598-024-53899-z

**Published:** 2024-02-08

**Authors:** Rut Romero-Martín, Isabel Caballero-Leiva, Maria Carmen Llasat, Montserrat Llasat-Botija, Tomeu Rigo, Herminia I. Valdemoro, Joan Gilabert, Maria Cortès, José A. Jiménez

**Affiliations:** 1 Laboratori d’Enginyeria Marítima, Universitat Politècnica de Catalunya·BarcelonaTech, c/Jordi Girona 1-3, Campus Nord ed. D1, 08034 Barcelona, Spain; 2 GAMA, Department de Física Aplicada, Universitat de Barcelona, 08028 Barcelona, Spain; 3 Servei Meteorològic de Catalunya, C. Berlin, 38-46, 08029 Barcelona, Spain; 4 Institut Cartogràfic i Geològic de Catalunya, Barcelona, Spain; 5 Delegació Territorial de Catalunya, AEMET, Barcelona, Spain

**Keywords:** Natural hazards, Ocean sciences

## Abstract

Coastal risks in the Mediterranean are a result of the complex interplay between hydrometeorological and marine hazards. The region encompasses areas with varying degrees of vulnerability to these hazards, as well as spatial variations in exposure values, making it essential to adopt a comprehensive and nuanced approach to risk assessment and management. It is worth noting that hydrometeorological hazards, such as flash floods, can often have a greater impact than strictly coastal hazards, highlighting the need to consider the full range of potential risks. Therefore, coastal managers must adopt a multi-hazard approach to make sound risk management decisions. This study addresses this need using an index-based framework that assesses the integrated risk in time and space (hereafter referred to as cumulative compound risk) in coastal zones by aggregating the main hydrometeorological and marine hazards, the vulnerability of the territory to both types of hazards, and values at exposure. The framework is designed for use at large spatial scales (applied to a 1100 km coastline in this study), with the basic spatial unit being relevant for management (here set as the municipality in this study). Its application enables the assessment of spatial variations in integrated risk as well as individual hydrometeorological and marine contributions. The combined use of the indices and cluster analysis helps identify similarities and differences in the risk profile of spatial units, and thus, define homogeneous areas from a risk management perspective. In this study, the framework was applied to the Spanish Mediterranean coastline, an area representative of the climatic, geomorphological, and socioeconomic conditions of the Mediterranean coast.

## Introduction

The Mediterranean coast, particularly the Western basin, is a heavily developed region that has experienced a significant concentration of tourism-driven population and economies. As a result, the coastal zone has become home to a dense network of infrastructure, assets, and values that are at high risk of exposure^1^. Because of its location and climatology, its coastal zone is susceptible to frequent natural hazards from both land and sea domains surrounding the coastline. Thus, the area experiences frequent heavy rainfall, thunderstorms, windstorms, and severe weather events^2^ that lead to intense natural hazards such as flash floods acting inland^3^. On the other hand, the interaction between coastal dynamics and human activities, both inland and along the coast, has resulted in significant shoreline erosion^4–6^. This, together with the impact of storms, has caused significant damage along the entire coast^7,8^. The convergence of terrestrial and marine hazards in an area with large exposure values, makes the coast a high-risk hotspot ^9–12^. Additionally, it’s important to note that these multiple drivers and/or hazards can occur simultaneously, resulting in a combination known as compound weather and climate events^13^.

Zscheischler et al.^14^ proposed a classification of compound events that offers a useful framework for analysing the mechanisms underlying their impact, and for developing risk adaptation strategies. In this study, we focus on what they called multivariate, spatially, and temporally compound events. A multivariate event occurs when hazards from multiple climate drivers co-occur in the same area, such as the case of compound coastal flooding caused by the co-occurrence of marine (coastal storm) and terrestrial (rainfall or river flow) drivers acting in the same site. Spatially compound events refer to co-occurring hazards from different climate drivers within a time window acting on different areas, leading to impacts that accumulate at spatially distant locations. Finally, temporally compound events occur due to a succession of hazards affecting a geographical region, which can lead to or amplify the impact compared to a single hazard. All these types of compound events are relevant from the risk management standpoint, as they can overwhelm the capacity of emergency response services. This can stretch the resources of emergency services beyond their limits, making it difficult to respond effectively to all the affected areas. Notable examples of this have been observed during the November 1966^15^ and 2018^16^ storms in Italy and the January 2020 Gloria^17,18^ storm in Spain. The occurrence of such events may be aggravated by climate change, as the rates of change observed in the Mediterranean basin exceed global trends for most variables^19^. Thus, climate change is expected to increase the intensity of coastal hazards in the area owing to sea-level rise (SLR)-induced erosion^6^, inundation^20^, and an increase in storm-induced flooding^21^ and heavy precipitation events^22^ among other effects. If we include the projected increase in coastal exposure^23^ as an influencing factor, the overall risk in the Mediterranean coastal zone is expected to increase in future decades.

To tackle the challenge of analysing compound events, some guidelines have been proposed^24^, one of which involves the use of event-based storylines. Event-based storylines are a way of analysing and communicating the impacts of extreme weather and climate events, with a focus on plausibility rather than probability^25^. They provide information on the underlying causes of the event, and are particularly useful in disaster risk management as they allow stakeholders to create hypothetical scenarios and conduct stress-test to improve emergency preparedness. In our case study, we have adopted this approach and customized it to fit our needs. Our assumption is that the overall risk in the area is a result of the combined impact of terrestrial and marine-induced hazards. This worst-case scenario takes into account the possibility that areas with high levels of high hydrometeorological and marine risks will also experience a high compound risk, which we refer to as cumulative compound risk in the coastal zone. To provide context for this approach, it is important to note that the study area, which encompasses the Spanish Mediterranean coast, is prone to relatively frequent multivariate and spatially compound heavy rainfall and coastal storm events^26^.

As a result of this, it is essential to adopt a comprehensive and effective disaster risk management strategy that can effectively account for the complex interplay between various hazards. One approach to achieving this is to identify and characterize coastal hotspots that are at high risk for cumulative compound events. This enables stakeholders to focus their efforts on the areas that require the most attention and to develop targeted mitigation and preparedness measures to minimize the impact of potential disasters. In this context, index-based risk and vulnerability assessments have become widely used tools that adopt different methodologies and approaches^27,28^. Many of the current index-based coastal vulnerability and risk assessments in the Mediterranean region focus exclusively on marine-induced hazards, with little consideration given to hydrometeorological hazards^29–31^. In cases where hydrometeorological hazards are included, they are often integrated within the same indicator, resulting in an overall value that fails to capture the unique characteristics of each hazard^32^. This approach can limit the accuracy and precision of the risk assessment, making it difficult to develop effective disaster risk management strategies. A more nuanced approach, such as the event-based storyline approach we are adopting, can provide a more comprehensive understanding of the complex interplay between different hazards, enabling stakeholders to make better-informed decisions and take more targeted action to mitigate risks.

Within this context, this study aims to present an index-based framework to assess the cumulative compound risk in coastal zones by aggregating the main hydrometeorological and marine hazards, vulnerability of the territory to both types of hazards, and exposure of the socioeconomic system. The framework can assess the individual contributions to risk of both domains (hydrometeorological and marine), as well as the relative contribution of each risk component (hazard, vulnerability, and exposure). It has been developed considering the specificities and needs of the Mediterranean coast, although it can be adapted to other regions by considering additional hazards and including other variables relevant to the local conditions of risk components. The framework is applied to the Spanish Mediterranean coast (northwest (NW) Mediterranean), which is subjected to the impact of flash floods, coastal storms, and compound events, and has spatial heterogeneity in coastal geomorphology and exposure representative of a large part of the Mediterranean coastline. Table 1 shows the main weather and climatic drivers and hazards considered in this study, while the variables used to indicate them are shown in Table 2 and described in the “Methods” section.

**Table 1:** Marine and hydrometeorological (terrestrial) drivers and hazards that act in each domain and control the cumulative compound risk in the coastal zone relevant to the study area.

Domain	Driver	Hazard
Marine	Coastal storms	Erosion-flooding
	Littoral dynamics	Mid-term erosion
	Sea level rise	Long-term erosion-inundation
Terrestrial/hydrometeorological	Rainfall	Flash flood—river flood—bad weather
	Thunderstorms	Flash floods
	Wind	Windstorms

**Table 2:** Data used to characterize marine and hydrometeorological hazards, vulnerability and coastal exposure, and for index validation.

Variable	Source	Data period
	Hazard	
Marine		
Significant wave height	SIMAR wave hindcast data base (Puertos del Estado)	1958–2018
Shoreline evolution rate	Aerial photographs (Institut Cartogràfic i Geològic de Catalunya (ICGC); Institut Cartogràfic Valencià (ICV))	1995–2018
Low elevation zone slope	2 m × 2 m Digital Elevation Models (ICGC; ICV)	2014, 2010
Shoreface slope	Global database^40^ plus local modifications using available local bathymetries	
Sea level rise	REDMAR Tidal gauge network (Puertos del Estado), IPCC AR6 regional projections^41^	1992–present
Hydrometeorological		
Thunderstorms	Radar network of the Meteorological Service of Catalonia (SMC) and lightning network of the Spanish State Meteorological Agency (AEMET)	2000–2018
Maximum wind gust	Daily wind data from automatic weather stations (AEMET)	1981–2015
Rainfall	Daily precipitation from automatic weather stations (AEMET)	1981–2015
	Vulnerability	
Marine		
Geomorphology	Aerial photographs (ICGC; ICV)	2018
Beach width	Aerial photographs (ICGC; ICV)	2018
Accommodation space	Aerial photographs (ICGC; ICV)	2018
Hydrometeorological		
Soil permeability	Spanish Land Use Information System—SIOSE (National Centre for Geographic Information (CNIG))	2014
Terrain slope	200 m × 200 m Digital Terrain Model (CNIG)	2015
Stream order	Hydrographical Classification of Rivers (Spanish Centre for Hydrographic Studies (CEDEX)	2016
	Exposure	
Land use	Spanish Land Use Information System—SIOSE (CNIG)	2014
Transport network	Aerial photographs (ICGC; ICV)	2018
Gross Household Disposable Income	Statitiscal Institut of Catalonia (IDESCAT); Generalitat Valenciana	2018
Economic activity	Tourism indicators. IDESCAT; Generalitat Valenciana	2018
Population	IDESCAT; Generalitat Valenciana	2018
	Validation	
Cumulative damage	Compensatory payments by the Consorcio de Compensación de Seguros (CCS)	1994–2021
Damaging floods frequency	INUNGAMA database^42^	1981–2020
Intensity of actual coastal damages	State of the Coastal Zone in Catalonia^43^	2000–2010
Intensity of Gloria-storm induced coastal damages	Compilation from different sources	2020

It’s worth noting that while storm surges are typically included in the analysis of compound coastal flooding^33^, we chose not to include them in our study because they are of very low amplitude in the study area^34^ and show little spatial variation along the coast. In other words, incorporating storm surges into our analysis would not have improved our characterization of coastal risk. Instead, we focused on wave-induced runup during storms, which is of much greater magnitude than storm surges in our study area, and is considered the main contributor to storm-induced coastal flooding^34^. Our approach is consistent with recent studies in the western Mediterranean that have used wave-induced runup and rainfall to characterize typical compound events in this region^26^. However, it’s important to note that storm surge could be included as an additional variable contributing to storm-induced marine hazards in areas where its magnitude is significant and/or where there is spatial variability. By taking a flexible and adaptable approach, disaster risk management strategies can be tailored to the specific hazards and drivers of risk in each region, resulting in more effective and targeted decisions to manage the impact of disasters.

## Methodological framework

In the context of this study, risks result from the dynamic interaction between weather-related hazards (of marine and hydrometeorological origin) modulated by the vulnerability of the physical system (both coastal and inland) and the exposure of the affected human system in the coastal zone. This is simply formulated as the product of the three components, *R* = *H · V · E*.

**Figure 1:**
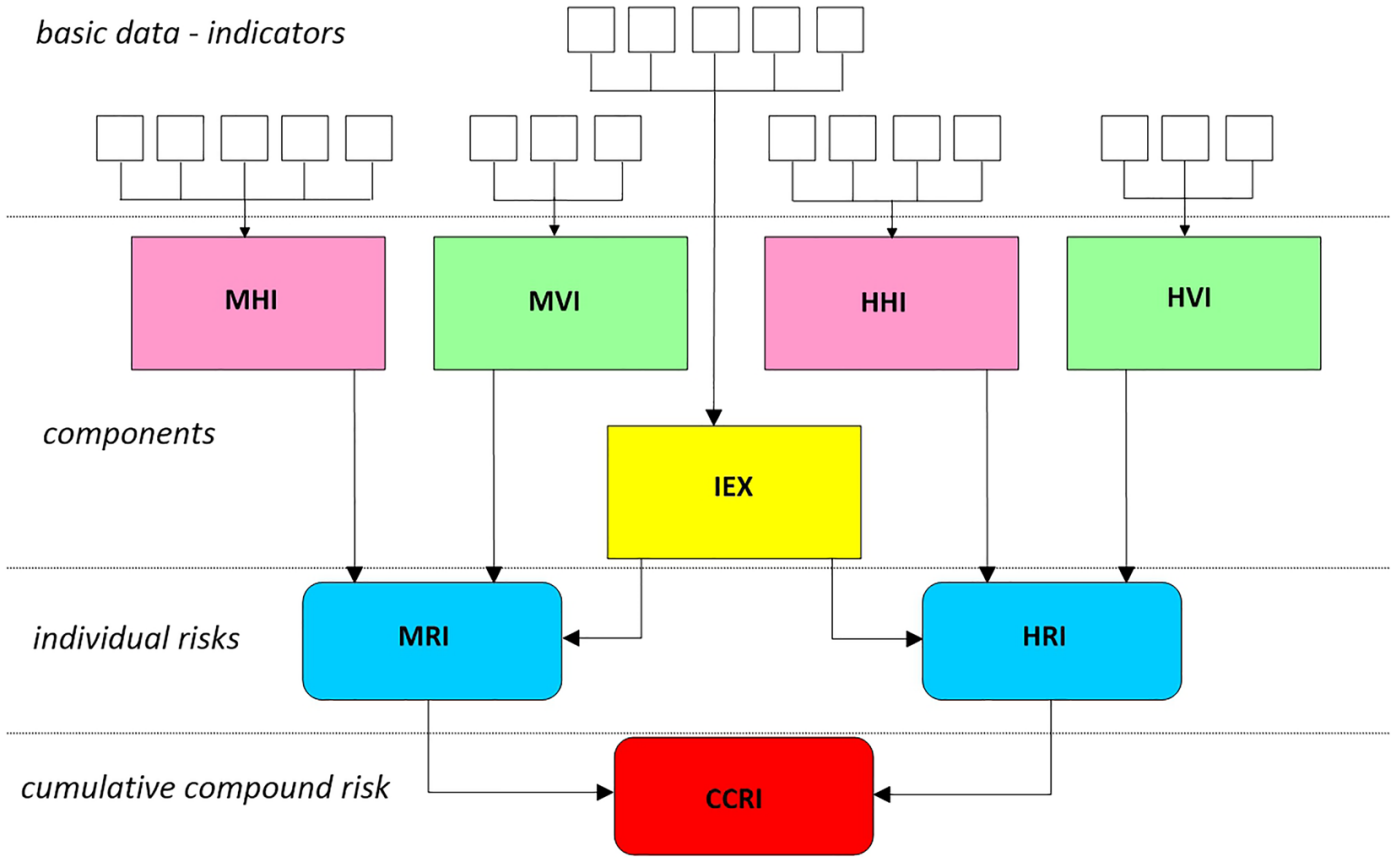
General framework to assess the cumulative compound risk index (CCRI). *MHI* marine hazard index, *MVI* marine vulnerability index, *HHI* hydrometeorological hazard index, *HVI* hydrometeorological vulnerability index, *IEX* exposure index, *MRI* marine risk index, *HRI* hydrometeorological risk index.

The methodological framework adopted in this study is shown in Fig. 1, where the risk is first calculated separately for the marine and hydrometeorological components (MRI and HRI, respectively) and then integrated into a cumulative compound risk index (CCRI). The hazard and vulnerability sub-indices are formulated individually for each component, while the exposure index (IEX) is common to both, as it serves to characterise the values at exposure where both hazards act, the coastal fringe. The methodology adopted to assess the cumulative compound risk and all contributing components is described in detail in the “Methods” section.

The indices used in this study were formulated following the generic form I = (I_1_ I_2_ … I_n_/n)^½^, where *n* is the number of components contributing to the index. The indices were calculated for coastal segments at the smallest possible scale to obtain the best spatial characterization of the analysed component. In the case of the marine domain, it was determined by the spatial heterogeneity of the geomorphology along the coast (2181 coastal segments along the entire coastline were defined), and subsequently integrated up to a spatial unit that characterizes the basic management scale, which was set as the municipality for this study (129 units). In the case of the hydrometeorological domain, all variables were considered at the municipality scale. All indices were classified into five class intervals, using a scale of 1 to 5, which, in qualitative terms, were classified from very low (VL) to very high (VH) for values 1 to 5, respectively.

The coastal risk calculated using a given index is the result of applying a given equation and an associated scale. However, this does not necessarily imply that the values obtained reflect the real risk of the area; it reflects the relative magnitude of the variables used to characterise it along the coast. To address this uncertainty, we validated the proposed index for the study area. Because cumulative compound risk integrates the consequences of all hazards acting at different time scales, selecting a single variable that adequately reflects it on the ground is challenging. To address this concern, we performed a double validation using different types of data to characterise the damage. Details on the validation are provided in the “Methods” section.

Finally, coastal municipalities were grouped according to similar attributes of marine and hydrometeorological risks that can be used to define coherent management strategies. To this end, coastal municipalities were classified into five clusters using K-means with three seeding variables: marine and hydrometeorological hazards and vulnerabilities, and exposure along the coast.

## Area of study and data

### Area of study

**Figure 2:**
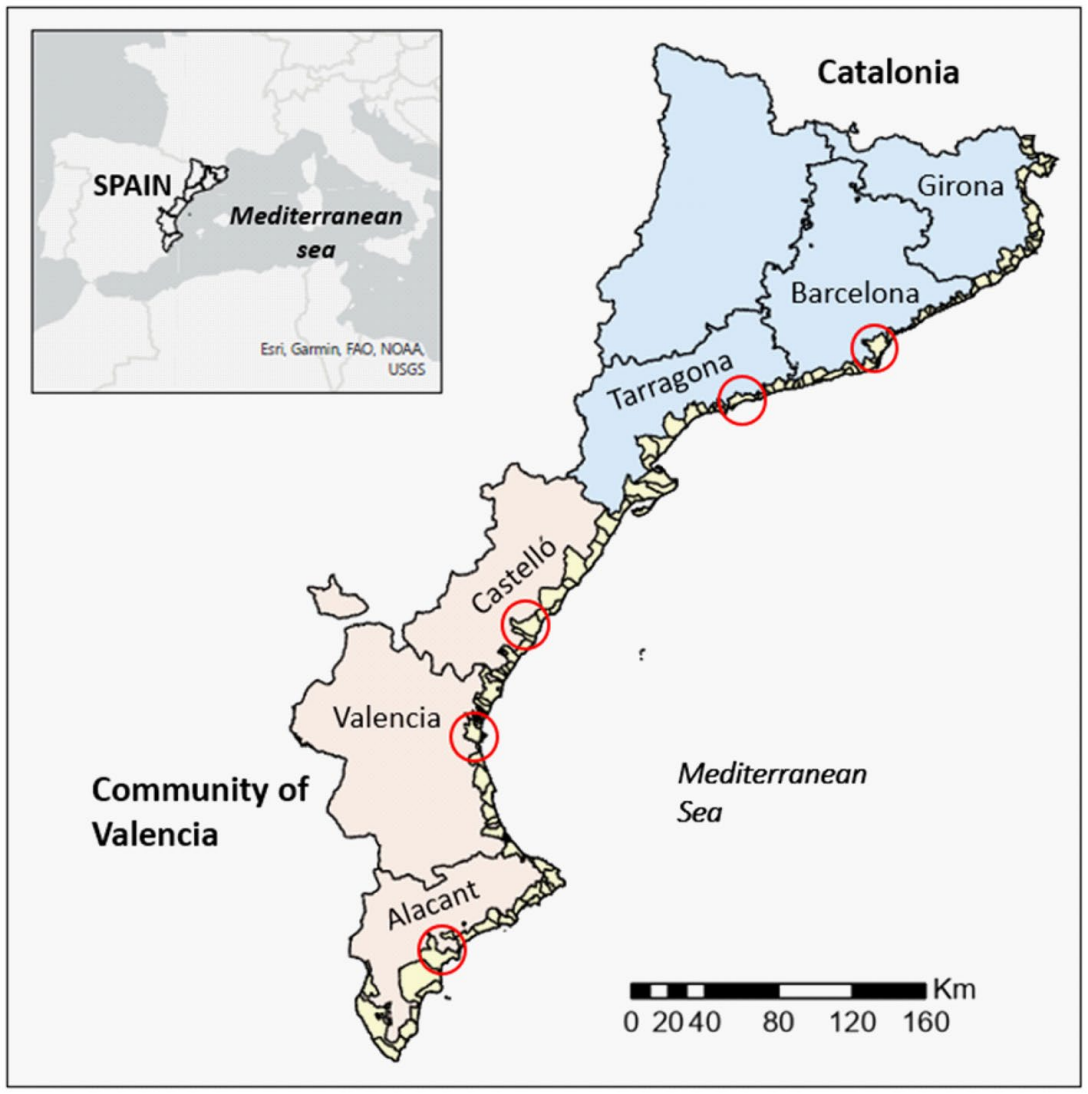
Map of the study area. Circles indicate the location of the capitals of each province and they correspond to the largest urban agglomerations, the cities of Barcelona, Tarragona, Castelló, Valencia and Alacant (by decreasing order of population). This map was created with Esri ArcGIS Pro 3 software (https://www.esri.com).

The study area extends along approximately 1100 km of the Spanish Mediterranean coast, and includes 129 municipalities in six provinces of two autonomous regions, Catalonia and Valencia (Fig. 2). It concentrates approximately 43% (3.23 M inhabitants) of the population of Catalonia and approximately 52% (2.6 M inhabitants) of the population of Valencia. Existing socio-economic activities are characteristic of Mediterranean coastal areas and are based on tourism, commerce, and agriculture^35^, with sun-and-beach tourism being the dominant activity^36^ that has led to the concentration of population and economic activities along the coastal fringe and surroundings over the last decades^37^.

From a geomorphological perspective, the Spanish Mediterranean coastline presents a great diversity of coastal habitats, such as cliffs, rocky coasts, sandy beaches, coastal plains, estuaries, river deltas, and stretches of artificial coasts maintained by beach nourishment and protected by coastal structures^38^. The area is subject to natural hazards, such as shoreline erosion, coastal storm impacts, and flash floods, which have produced significant impacts and damage along the coastal zone^3,5,8–11,17,18,39^.

### Data

The data used in this study can be grouped into those used to characterise hazards, vulnerability, and exposure and are listed in Table 2. All data were collected at the smallest available spatial scale according to the needs of each indicator (see the “Methods” section).

## Results

The results of the application of the proposed methodology consist of an assessment of the cumulative compound risk along the Spanish Mediterranean coast, the marginal marine and hydrometeorological risks, and the contribution of each risk component, that is hazard, vulnerability, and exposure.

### The marine domain

**Figure 3:**
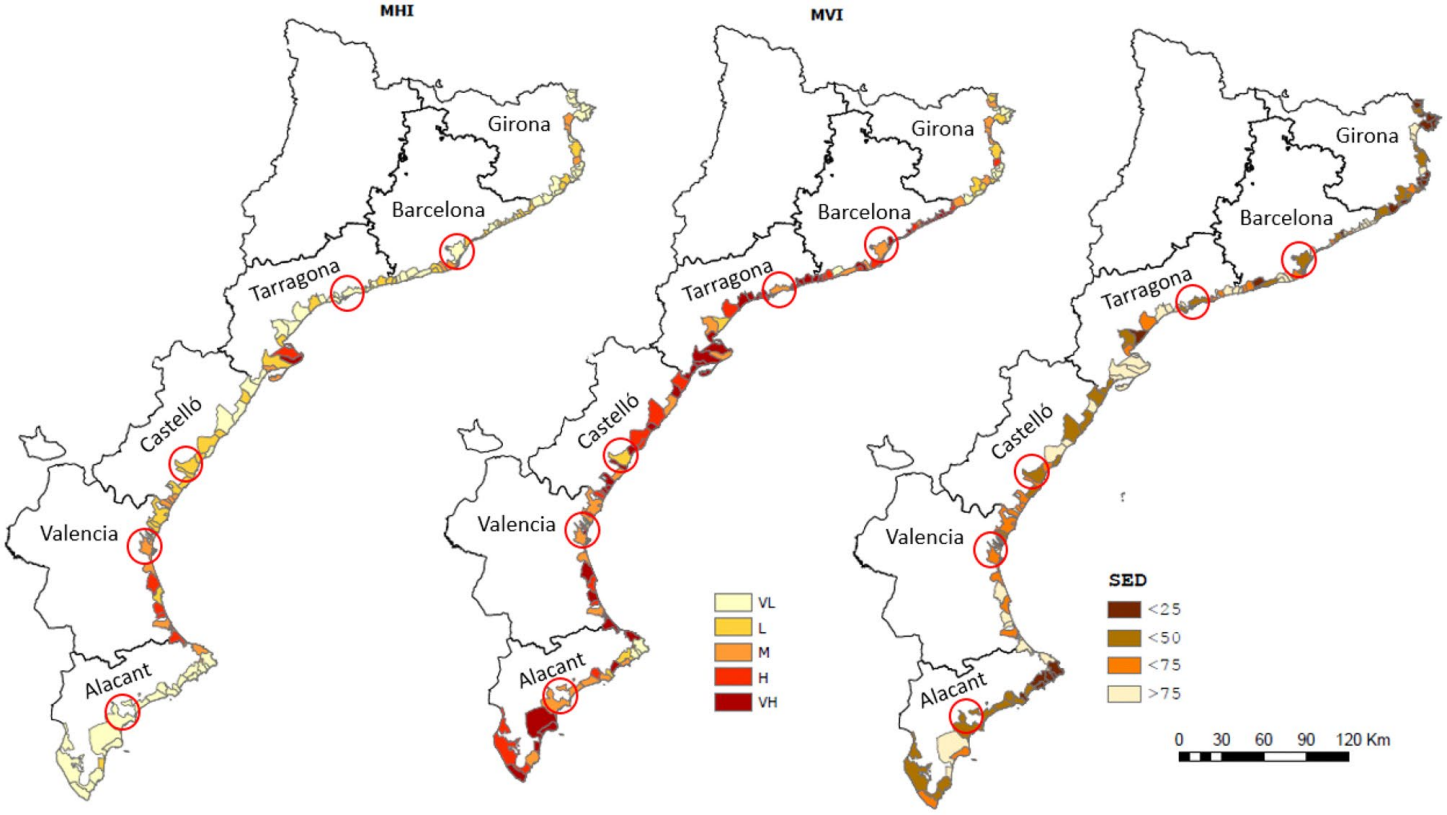
Marine hazard (3a, left), and vulnerability (3b, middle) indices; and percentage of sedimentary coastline with respect to total shoreline within each municipality (3c, right). (*VL* very low, *L* low, *M* medium, *H* high, *VH* very high). Circles indicate the location of the capitals of each province, if situated on the coast. These maps were created with Esri ArcGIS Pro 3 software (https://www.esri.com).

Figure 3a shows the spatial distribution of the marine hazard index integrated at the municipal level along the study area, and the statistics of municipalities per hazard class are shown in Table S1 (Supplementary Information). In general, coastal municipalities along the study area are subjected to a relatively low number of hazardous conditions, with only approximately 8% of the municipalities being classified as having a high or very high hazard level. However, this result should be carefully considered as these values integrate all segments within a given municipality and, in many cases, a large percentage of the coastline consists of rigid features (rocks, cliffs, and structures) which have a very low associated hazard value. When integrated with sedimentary segments within the municipality, their relative weights may dominate the final hazard value. To assess the potential importance of this factor, the percentage of rigid shoreline for each municipality along the study area was considered (Fig. 3c), which shows that areas with larger rigid coastlines are usually those subjected to low hazardous conditions.

The percentage of shoreline length of the study area that belongs to each class with respect to the total coastline for all hazard indicators is shown in Fig. S2 (Supplementary Information). The results show that storms are the most important contributors to the most hazardous conditions, whereas shoreline evolution (with the exception of sea-level rise (SLR)) is the lowest owing to the large percentage of non-erodible coastlines. It must be stressed that the values associated with the SLR are dependent on the selected scenario, and this study used actual SLR values. This method of assessing marine hazards provides integrated values at the municipality scale and allows for smaller scale values (segments along the coast) to be retained for more detailed analysis if necessary.

The spatial distribution of the coastal vulnerability integrated at the municipal level is shown in Fig. 3b, and the statistics of the municipalities per class are shown in Table S1 (Supplementary Information). As shown in Fig. 3b and Table S1, coastal municipalities with high and very high vulnerability predominate throughout the study area, with nearly 60% of the municipalities belonging to these classes. As expected, the municipalities with low vulnerability correspond to those wherein the percentage of rigid coastline predominates (Fig. 3c). The coastal geomorphology and the lack of accommodation space are the main factors responsible for stretches with the highest vulnerability in the study area. The percentage of the shoreline length of the study area that belongs to each class for all vulnerability indicators is shown in Fig. S3 (Supplementary Information). The difference in the frequency of high vulnerability and hazard values along the coast would indicate that high-risk conditions in the area are driven more by the high sensitivity of the coast to the hazards under consideration than by the magnitude of the hazards.

### The hydrometeorological domain

**Figure 4:**
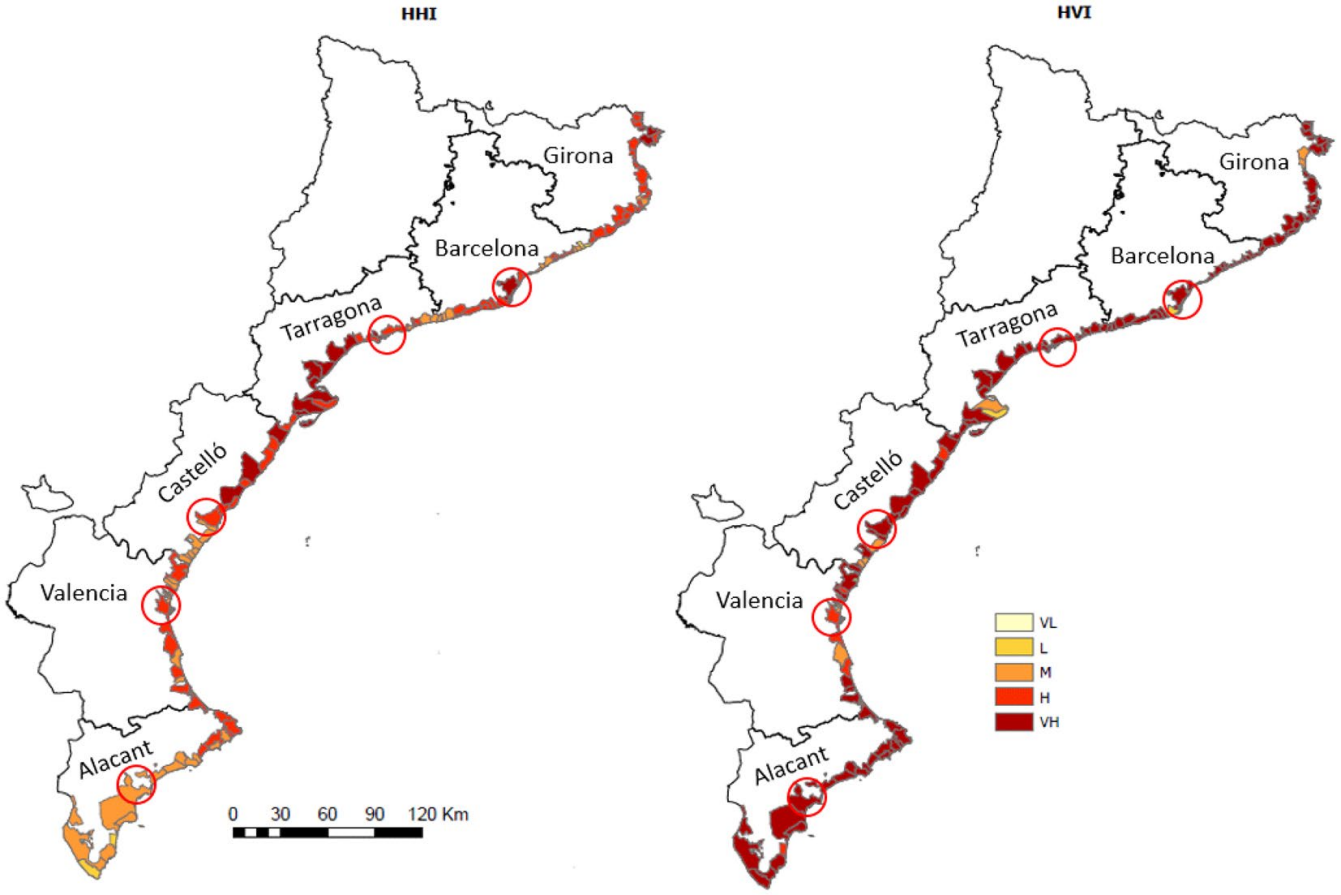
Hydrometeorological hazard (left) and vulnerability (right) indices along the study area. (*VL* very low, *L* low, *M* medium, *H* high, *VH* very high). Circles indicate the location of the capitals of each province, if situated on the coast. These maps were created with Esri ArcGIS Pro 3 software (https://www.esri.com).

Coastal municipalities along the study area are subjected to high hydrometeorological hazardous conditions, with approximately 60% of the municipalities being classified as having high or very high hazard (Fig. 4, Table S2 in Supplementary Information). Among the variables considered to determine the hazard, wind is the lowest contributor to the overall hydrometeorological hazard in the area, with approximately 75% of the municipalities being ranked as having low or very low intensity (Fig. S4 in Supplementary Information).

The study area can be also classified as highly vulnerable to hydrometeorological hazards, with 80% of coastal municipalities being classified as having very high vulnerability (Fig. 4, Table S2 in Supplementary Information). This is consistent with the large occurrence of damaging flood events in the area over the last decades^10,44^. Among the variables considered to determine the vulnerability, the stream order is the largest contributor to the overall vulnerability (Fig. S5 in the Supplementary Information), presenting a relatively homogeneous distribution owing to the typical structure of river courses in the study area, mostly characterised by small catchment basins.

### Exposure

**Figure 5:**
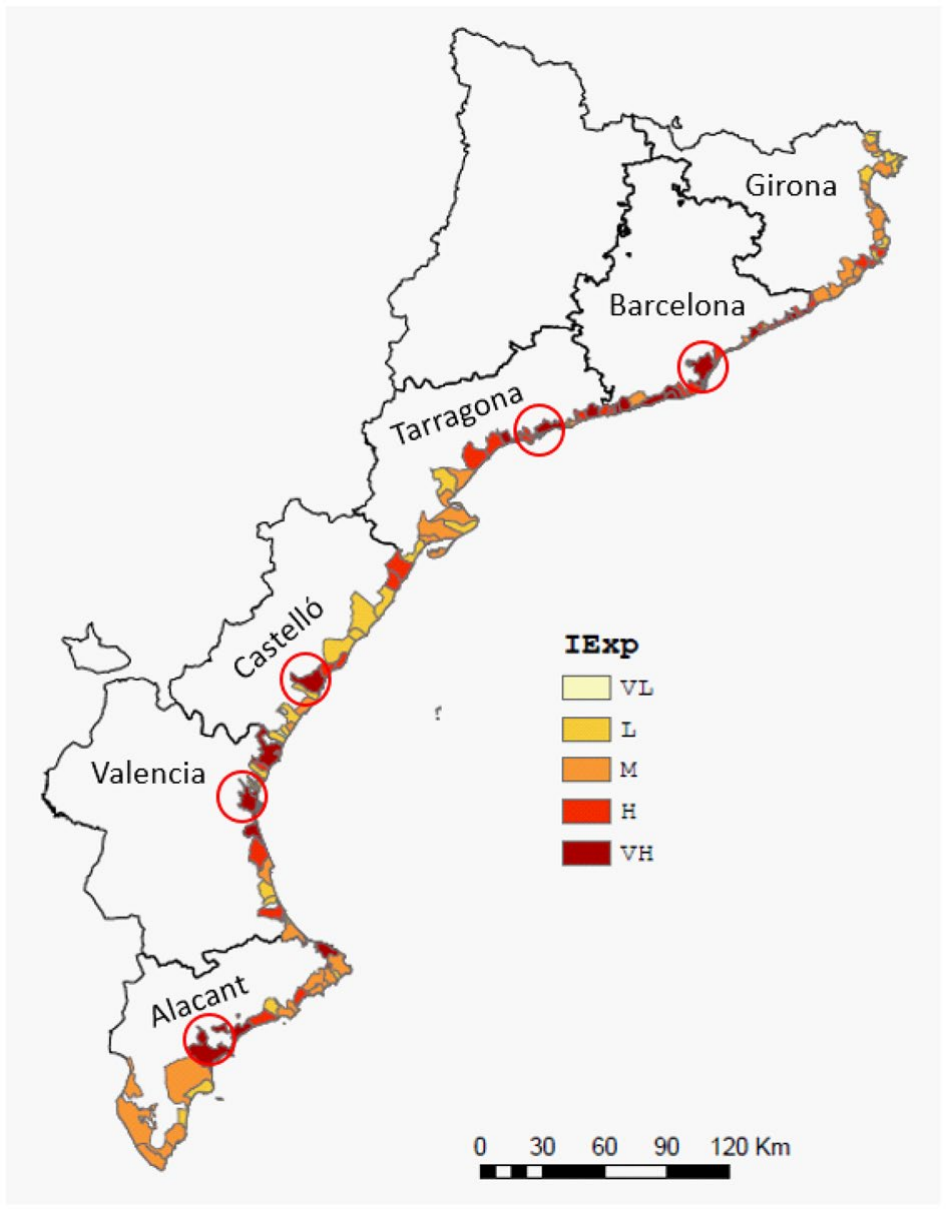
Coastal exposure index along the study area. (*VL* very low, *L* low, *M* medium, *H* high, *VH* very high). Circles indicate the location of the capitals of each province, if situated on the coast. This map was created with Esri ArcGIS Pro 3 software (https://www.esri.com).

The exposure values obtained for the study area are representative of a highly developed coastal zone, such as the Mediterranean, where the existence of a relatively large human concentration and its associated activities implies that approximately 42% of the municipalities have high and very high exposure, which, with the inclusion of the medium class, increases to 73% of the municipalities (Fig. 5). Notably, because land use and transport networks are the only two indicators classified in absolute terms (the other three are classified according to the quintile method and consequently reflect relative local conditions), they are the main factors that define the final variability in exposure conditions. This is evident in the distribution of high and very high classes that correspond to large urbanised areas around main cities, such as Barcelona, Tarragona, and Valencia. The percentages of municipalities belonging to each exposure class for each exposure indicator are shown in Fig. S6 (Supplementary Information).

### Cumulative compound coastal risk

**Figure 6:**
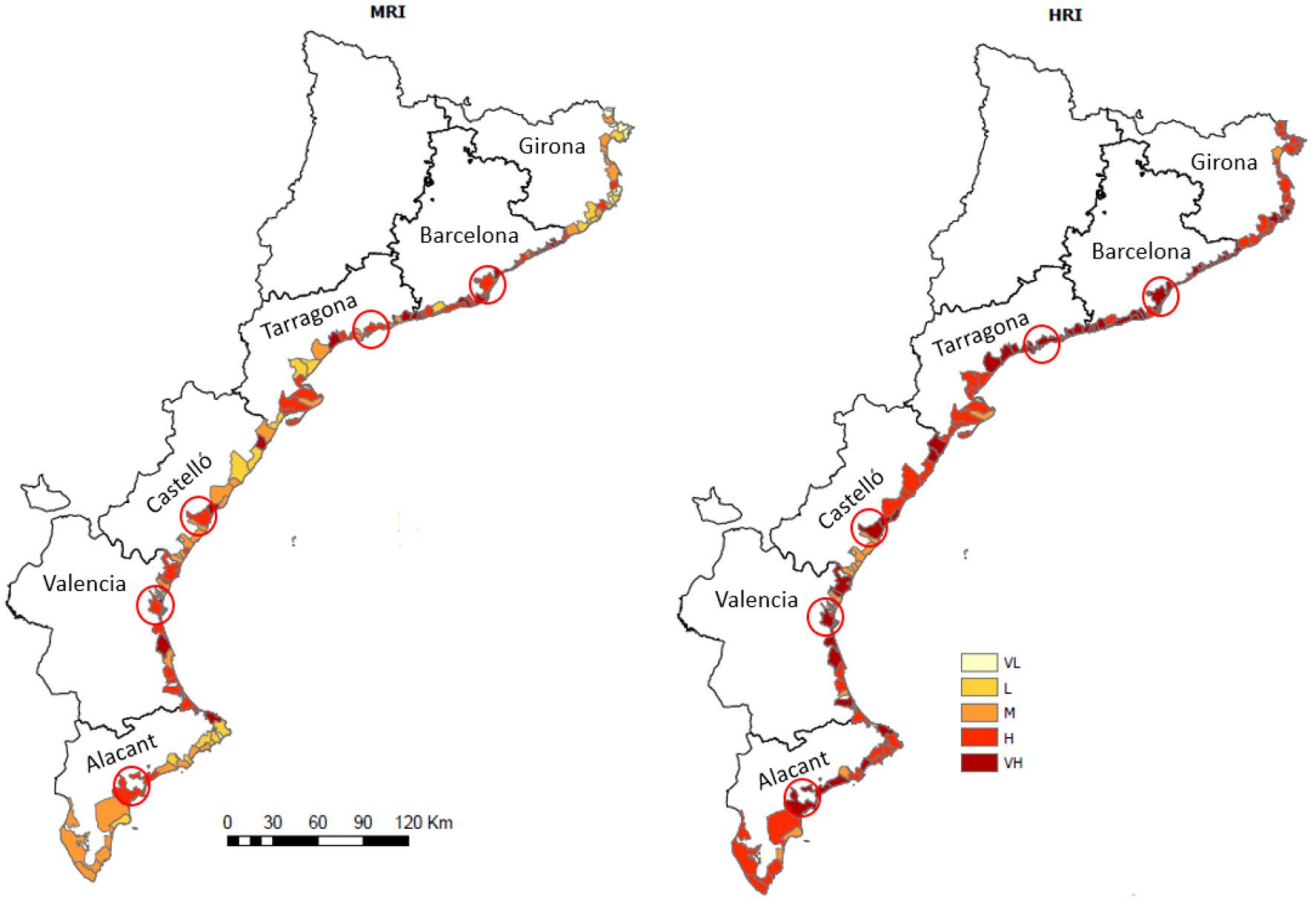
Marine (left) and hydrometeorological (right) risk indices along the study area. (*VL* very low, *L* low, *M* medium, *H* high, *VH* very high). Circles indicate the location of the capitals of each province, if situated on the coast. These maps were created with Esri ArcGIS Pro 3 software (https://www.esri.com).

The highest risk conditions in the area are associated with the hydrometeorological component (HRI), with 82% of the municipalities belonging to high or very high-risk classes, whereas for marine-induced risks (MRI), these classes amount to 49% (Fig. 6, Table S3 in Supplementary Information). This result clearly indicates that any risk management strategy applied in this coastal zone cannot only address marine hazards but, on the contrary, has to adequately include the management of hydrometeorological-induced risks. This dominance is consistent with the findings of previous studies in the study area that identified flash floods as more damaging than coastal storm-induced flooding^45^.

**Figure 7:**
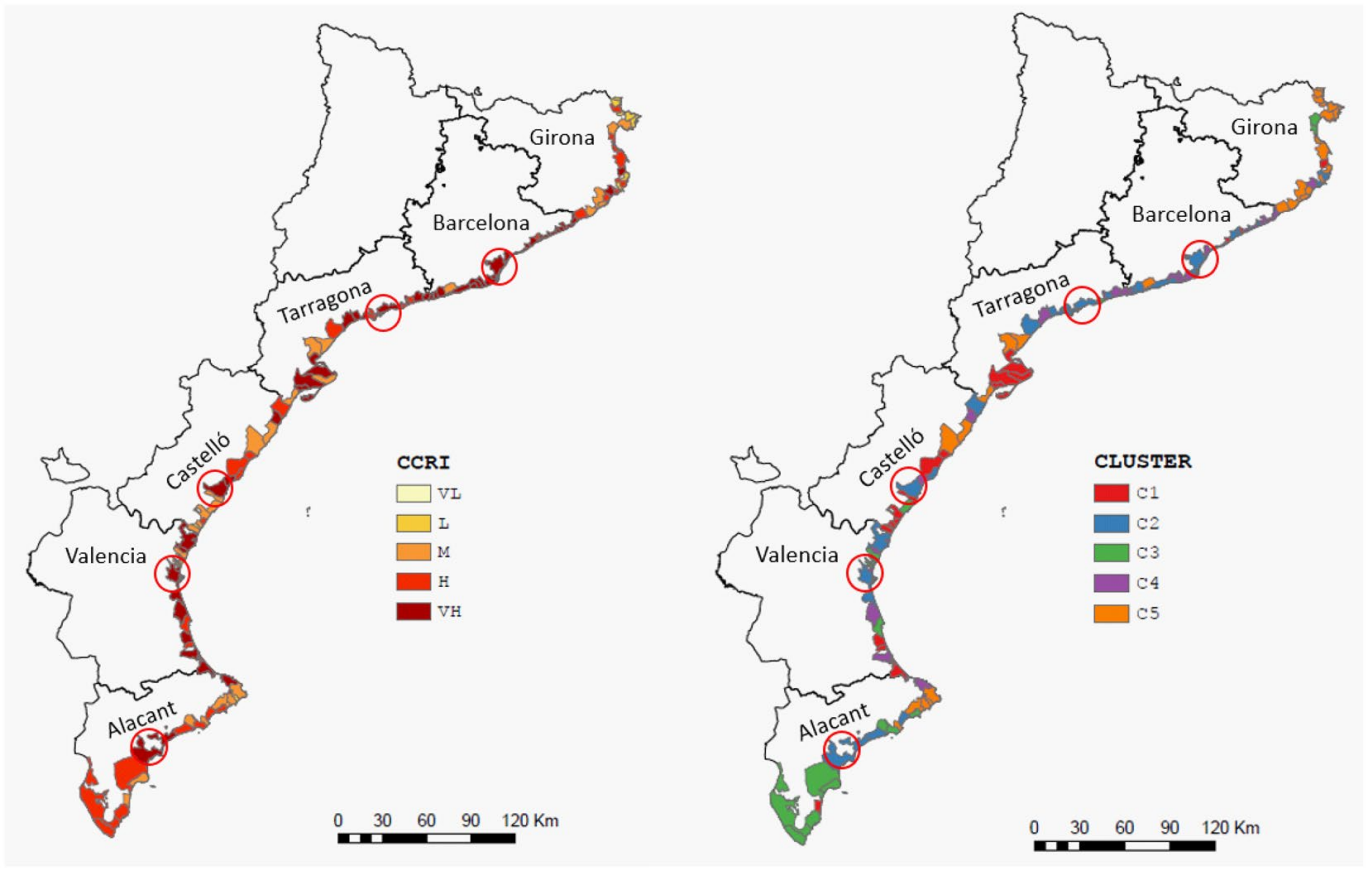
Cumulative compound coastal risk (left) and classes of municipalities clustered in terms of their marine and hydrometeorological hazard and vulnerability, and exposure at the coastal zone (right). (*VL* very low, *L* low, *M* medium, *H* high, *VH* very high). Circles indicate the location of the capitals of each province, if situated on the coast. These maps were created with Esri ArcGIS Pro 3 software (https://www.esri.com).

When both type of risks are combined to evaluate the cumulative compound risk, the area is characterised by the dominance of high-and very high-risk conditions, with 26% and 43% of the municipalities falling into these categories, respectively (Fig. 7). This reflects one of the main characteristics of the NW Mediterranean coastal zone, where a combination of natural and socioeconomic conditions determines high-risk conditions^20^.

Once partial and compound risk indices were calculated for all coastal municipalities, cluster analysis was applied to identify the existence of a spatial pattern underlying the values obtained, beyond the identification of sites with a higher or lower risk. Thus, the 129 municipalities in the area were grouped into five clusters, the main representative characteristics of which are shown in Table 3 and Fig. S7 (Supplementary Information). The first aspect to highlight is that, despite using the same seeding information, the clusters present a much clearer spatial pattern (Fig. 7) than the CCRI, and in this sense, they can be considered as an additional element to identify risk conditions in the area and help define risk management strategies.

**Table 3:** Main characteristics of risk clusters.

Cluster	Municipalities % (N)	CCRI	MRI	HRI	IEX	MHV	HHV
C1	21.7 (28)	4.2	3.6	3.6	2.4	4.3	4.3
C2	20.9 (27)	4.6	3.5	5	4.6	2.0	4.7
C3	16.2 (21)	3.2	2.5	3.3	2.5	2.2	3.6
C4	20.9 (27)	5.0	4.8	4.9	4.2	3.8	4.4
C5	20.1 (26)	2.9	1.9	4.0	2.6	1.3	4.9

Values are given as the average of the corresponding values to all municipalities belonging to each cluster. (*CCRI* cumulative compound risk, *MRI* marine risk, *HRI* hydrometeorological risk, *IEX* exposure, *MHV* marine hazard·vulnerability, *HHV* hydrometeorological hazard·vulnerability, *%* percentage of municipalities within the study area, *N* number of municipalities).

Thus, there were two clusters (C4 and C2) that stood out significantly with the highest compound risk values (Fig. S7, Supplementary Information). The municipalities that comprise them either present a very high risk (C4) or are distributed between very high and high conditions (C2). Despite this similarity, they presented different risk profiles, with C4 having much higher individual risks than C2. This is owing to the lower marine risk of C2, where the main contributor is high exposure, whereas the joint contribution of hazard and vulnerability is low. Their spatial distribution is very well defined, and they are concentrated in the most developed areas, such as the entire province of Barcelona, the northern half of Tarragona, most of the Valencia coast, and the central part of Alacant (Fig. 7). All of these correspond to coastal areas with a high population and, consequently, are largely urbanised. This is clearly evidenced in the average values of the exposure index IEX, which presented the highest values for all clusters (Table 3). At the opposite extreme, cluster C5 comprised areas with the lowest compound risk, for which, although they present a high hydrometeorological risk, the final value is determined by low marine risk and exposure indices. They are mainly concentrated along almost the entire coast of the province of Girona, the area of the Cape north of Alacant, and two spots in Castelló and south of Tarragona (Fig. 7). All these sites are characterised by rocky coastlines. Finally, the two remaining clusters are distributed as spots along the entire study area, with C1 comprising municipalities with high risk but low exposure values and C3 with medium values for risks and exposure indices.

Spatially, Barcelona Province presents the highest compound risk as well as marine and hydrometeorological risk. It concentrates the highest exposure and approximately 93% of its coastline (22 of its 27 municipalities) belongs to the riskiest clusters (C2 and C4) (Table S4 in Supplementary Information). By contrast, Girona presented the lowest compound risk, with only 16% of its coastline (four of its 22 municipalities) belonging to the riskiest clusters (C2 and C4) (Table S4 in Supplementary Information). This occurs for a province with a high hydrometeorological risk, as the lowest assessed marine risk owing to its large percentage of rocky coastline offsets its contribution to compounding. The areas with the highest compound risk are located in municipalities with intensive tourism, which have high exposure values, and a large part of their coastline is made up of beaches. The main risk characteristics of all provinces in the study area are listed in Table S4 (Supplementary Information).

### Validation

**Figure 8:**
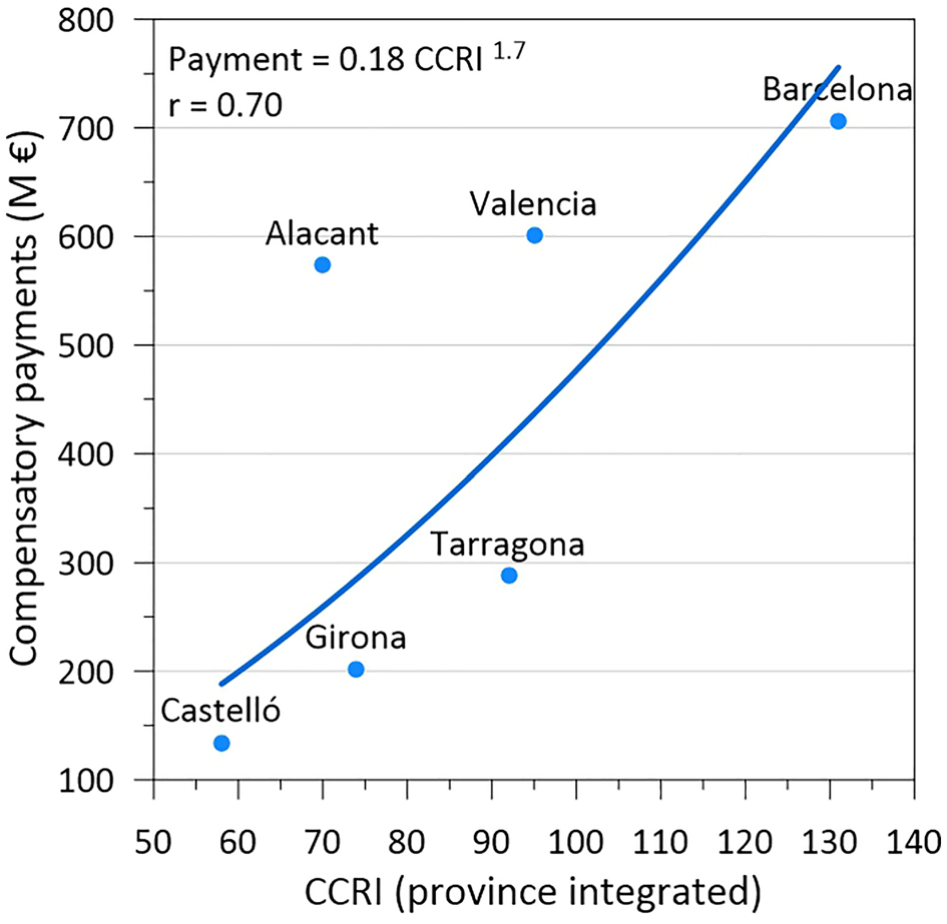
Compensatory payments by CCS for damages occasioned by extraordinary floods, sea storms, and atypical cyclones in the period 1994–2021 aggregated for the provinces of the study area versus the cumulative CCRI values (calculated as the sum of the indices for each coastal municipality for each province).

The methodology proposed in this study enables the mapping of cumulative compound risks along the Catalan coast. However, because this is the result of the calculation of an index (set of equations) and the application of an intensity scale, validating them is essential to increase managers’ confidence in the results obtained. As CCRI integrates the action of a variety of hazards and values of exposure, we performed two different validation exercises (see the “Methods” section). Figure 8 shows a comparison of the calculated risk index with data on compensation payments by the Consorcio de Compensación de Seguros (CCS) for the impact of floods, coastal storms (sea battering), and extraordinary atypical cyclones, both integrated at the province scale. As shown in Fig. 8, there is a relationship between payments and CCRI in a manner that increasing values of CCRI result in increasing values of payments. However, there are two provinces in which payments exceed the expected value according to their CCRI values. Notably, these payments only cover damages for properties that have been insured, and do not correspond to the actual damage but to the percentage covered by the CCS. In addition, they integrate all damage within the province, not only in coastal municipalities. Regardless, because of the concentration of assets and population in the coastal zone, we assume that they are proportional to the damage caused by the considered hazards.

Table 4 shows the results of the second qualitative validation, where we compared CCRI with qualitative damage values integrating hydrometeorological (floods) and marine impacts, the damage index (see the “Methods” section). If we focus on the largest damage classes, that is, those with a value ≥ 3 (M, H, VH), 93% of them correspond to municipalities with a CCRI ≥ 3 (M, H, VH). By contrast, if we focus on the largest CCRI classes, that is, those with a value ≥ 3 (M, H, VH), 98% of them correspond to municipalities with a damage index ≥ 3 (M, H, VH). In summary, both validation exercises indicate that the higher the CCRI, the greater the damage associated with both types of hazards. Consequently, CCRI can be considered a good predictor of the expected cumulative compound coastal risk for Mediterranean conditions.

**Table 4:** Number of municipalities along the Catalan coast classified in terms of the CCRI and damage index.

	CCRI					
		VL	L	M	H	VH
Damage index	VL	0	0	0	0	0
	L	0	1	1	0	0
	M	0	3	2	5	8
	H	0	2	7	9	23
	VH	0	0	0	1	8

## Discussion and conclusions

In this section, the proposed methodology and obtained results are discussed in terms of their applicability in coastal risk management. The proposed index, the CCRI, assesses the integrated contribution in space and time of marine and hydrometeorological hazards in a given spatial management-oriented unit, the municipality. It does not strictly reflect the risk associated with compound events, as described by Zscheischler et al.^14^, but characterizes the cumulative action of all contributing processes. Thus, the higher the CCRI, the greater are the risks associated with the different hazards at the site, and consequently, the greater is the importance of jointly considering them to adequately manage the overall coastal risk.

This method also retains the partial contributions of hydrometeorological and marine processes, which is relevant because their impacts and the way in which they are induced vary, and therefore, they should be managed differently. The approach adopted in this study defines risk in terms of three components, that is, hazard, vulnerability, and exposure, and this permits the identification of their relative contribution to the risk and, consequently, the investigation of risk reduction strategies addressing specific components.

**Figure 9:**
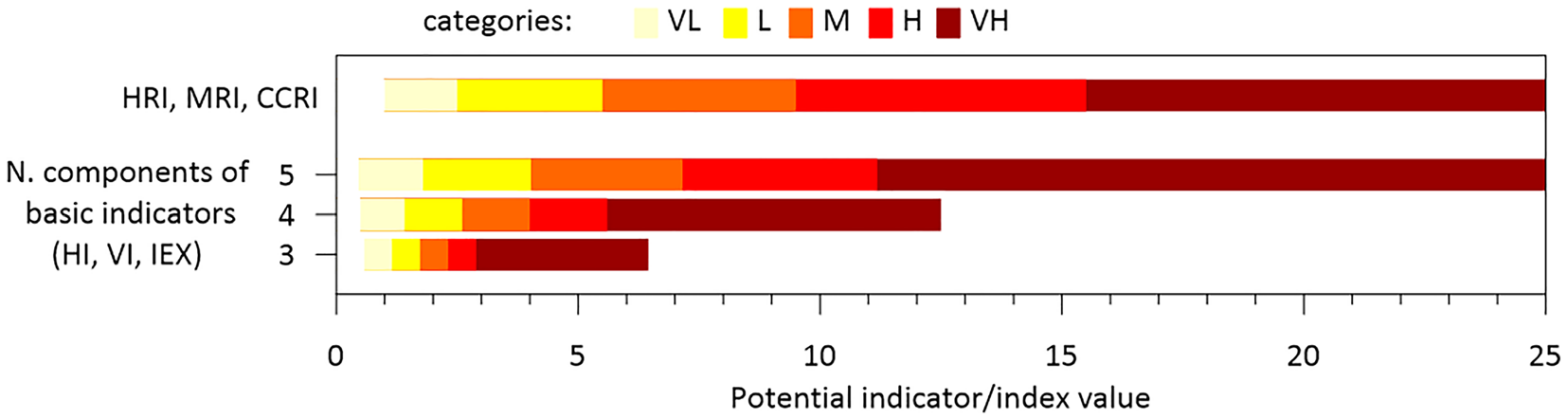
Scale for classifying and rescaling (i) basic indicators (hazard, vulnerability, exposure) as a function of the number of components composing them; and (ii) risk indices.

One of the main challenges with classifying indicators is the choice of scale, which determines whether they reflect the relative or absolute conditions of the process represented. A typical example of relative scaling is the quintile method, which is one of the most widely used methods in index-based analysis^46,47^. However, although it serves to compare areas with each other, it does not necessarily characterise actual vulnerability/risk conditions and does not permit comparison with analyses at other sites^48^. We opted to use absolute-oriented scaling in a manner that allowed us to simultaneously compare across sites and characterise the actual considered magnitude. The basic sub-indices (hazards, vulnerability, and exposure) were classified on a scale of 1 to 5 by imposing a criterion whereby if the majority of their components (four of five, three of four, and two of three) belonged to a given class, the value corresponding to that combination determined the lower end of that class (Tables 5,6, 7). This resulted in a nonlinear scale where, if most of the variables contributed to inducing a very high hazard, vulnerability, or exposure, a single low value would not partially counteract them (Fig. 9). In the case of risk indices, a nonlinear scale was also used (Fig. 9) which resulted from applying the combination rules presented in Table 8.

**Table 5:** Ranges determining the classes of variables and indices used to characterize marine hazards and vulnerability.

	VL	L	M	H	VH
	1	2	3	4	5
Hazard					
Hs (m)	< 3.2	[3.2, 4.0[	[4.0, 4.8[	[4.8, 5.9[	≥ 5.9
SEL (m/a)	> 0.0	[0.0, − 0.2[	[− 0.2, − 1.0[	[− 1.0, − 1.8[	≤ − 1.8
SF slope	> 0.04	[0.04, 0.02[	[0.02, 0.01[	[0.01, 0.009[	≤ 0.009
mLECZ slope	> 0.0097	[0.0097, 0.0074[	[0.0074, 0.0039[	[0.0039, 0.0024[	≤ 0.0024
SLR (m)	< 0.24	[0.24, 0.38[	[0.38, 0.66[	[0.66, 0.95[	≥ 0.95
MHI	< 1.8	1.8–4.0	4.0–7.2	7.2–11.2	> 11.2
Vulnerability					
Geomorphology	Cliff/rocky	Artificial/revetment	Erodible cliff/bluff	Gravel	Sand
Beach width (m)	> 64	[64, 48[	[48, 32[	[32, 16[	≤ 16
Acomm space (% of beach length)	> 75	[75, 50[	[50, 25[	≤ 25	No space
MVI	< 1.15	1.15–1.7	1.7–2.3	2.3–2.9	> 2.9

**Table 6:** Ranges determining the classes of variables and indices used to characterize hydrometeorological hazards and vulnerability.

	VL	L	M	H	VH
	1	2	3	4	5
Hazard					
NTD (no. days/year)	≤ 3	[3, 5[	[5.0, 10.0[	[10.0, 15.0[	≥ 15.0
MWG (km/h)	≤ 50	[50, 70[	[70, 90[	[90, 100[	≥ 100
NRDS (no. days/year)	≤ 10	[10, 15[	[15, 20[	[20, 30[	≥ 30
T20 (mm)	≤ 40	[40, 100[	[100, 200[	[200–300[	≥ 300
HHI	< 1.4	1.4–2.6	2.6–4.0	4.0–5.6	> 5.6
Vulnerability					
Soil permeability (runoff coefficient)	< 0.36	[0.36, 0.38[	[0.38, 0.41[	[0.41, 0.45[	≥ 0.45
Slope (degrees)	≤ 0.75	[0.75, 1.5[	[1.5, 3.0[	[3.0, 6.0[	≥ 6.0
Stream order	≤ 1.5	[1.5, 2.5[	[2.5, 3.5[	[3.5, 4.5[	≥ 4.5
HVI	< 1.15	1.15–1.7	1.7–2.3	2.3–2.9	> 2.9

**Table 7:** Ranges determining the classes of variables and index used to characterize values at exposure.

	VL	L	M	H	VH
	1	2	3	4	5
Exposure					
Land Use	Barren/Marshland, Grassland and Rockland	Forests and Parks	Beaches and Crops	Camping and Industry	Urban and Transport
Transport	No significant	Local road	National road	Motorway	Railway
GFDI (1000€/hab)	≤ 11.9	[11.9, 13.0[	[13.0, 13.9[	[13.9, 15.7[	≥ 15.7
Economic Activity (places)	≤ 583	[583,1830[	[1830, 5030[	[5030–10696[	≥ 10,696
Population (hab)	≤ 4130	[4130, 10585[	[10585, 20053[	[20053–33362[	≥ 33,362
IEX	< 1.8	1.8–4.0	4.0–7.2	7.2–11.2	> 11.2

**Table 8:** Rules for the reclassification of the product of two indices with values between 1 (VL) and 5 (VH). Resulting values shown in each cell inside the table (between 1 and 25) are reclassified on a scale of 1 to 5.

			Index 1				
			VL	L	M	H	VH
			1	2	3	4	5
Index 2	VL	1	1	2	3	4	5
	L	2	2	4	6	8	10
	M	3	3	6	9	12	15
	H	4	4	8	12	16	20
	VH	5	5	10	15	20	25

Another issue related to the scale is how the magnitude of the variables that compose the hazard, vulnerability, and exposure sub-indices is assessed. Variables contributing to the hazard, vulnerability, and exposure were classified using a scale referring to a well-defined target; although they reflect the characteristics of the study area, they can be easily adapted to local conditions elsewhere. For example, the medium-term erosion indicator was scaled with reference to the average beach width. Thus, the classes were defined according to the value required to erode a certain fraction of the average width. To apply it to another area or to fix a given management criterion, it is sufficient to change the reference value such that the scale is directly adjusted to local conditions while maintaining the same criterion. In other cases, variables were scaled using existing hazard scales for the study area (e.g., rainfall, winds, and storms), which can also be substituted by those existing in the area of application or if new risk warning criteria are set.

An important aspect usually not covered in the use of this type of indicator is its validation^27,49^. This is the only way to move from the results obtained by applying a set of equations and scale to the ground reality. In other words, we assess whether the indices measure what they are supposed to measure. In this study, we performed a double validation of the CCRI against two independent datasets representing the cumulative damage of the considered hazards in the coastal zone. Obtained results for aggregated values at the province scale indicate that CCRI is a good indicator of the cumulative compound risk, such that the larger the CCRI, the larger are the damage. The small-scale analysis shows a similar trend but with greater dispersion, which may be attributed to the qualitative nature of the information used.

Finally, the combined use of the indices and cluster analysis has proven to be a valuable approach for identifying spatial units that are homogeneous from the risk perspective. This allows for the identification of similarities and differences in the risk profiles of spatial units. From a management standpoint, it is clear that it is of high value to have both types of results to design more efficient risk-management strategies.

With respect to the study area, the cumulative compound coastal risk was dominated by the hydrometeorological component, which reached values significantly higher than those associated with the marine component. This agrees with the results of previous studies, where damage and impacts associated with flash floods have been identified as very important in terms of tangible damage and mortality^9,44^. It is necessary to consider that, while damages from marine-induced hazards are usually restricted to the outer coastal fringe and infrastructures therein^11,50^, hydrometeorological hazards impact a larger portion of the territory occupied by urbanized areas. In this sense, the increasing urbanisation in flood-prone areas permitted by local administrations during the last decades has largely shaped the current flood risk scenarios along the Spanish Mediterranean coast^51^.

With respect to the territory, the areas with the highest compound risk are concentrated in the most urbanised areas of the coastal zone, where the combination of the highest exposure, medium to high hazards, and vulnerability values dominate. This agrees with the findings of previous studies on river and flood damage along the Mediterranean Spanish coast that identified the dominant role of socio-economic factors in amplifying risks over climate-related factors^52,53^. Similarly, Jiménez et al.^8^ analysed the damage induced by marine storms along the Catalan coast during the second half of the twentieth century and found that, in the absence of an increase in storminess, the observed increasing trend could be explained by increasing values at exposure together with an increase in coastal vulnerability owing to decreasing beach widths.

The analysis suggests that urban development in the coastal zone is a significant contributor to the increase in risk. While climate change will exacerbate future risk levels, the increasing use of the term “climate emergency” to describe any extreme event affecting our coasts may obscure our role in creating this situation. It is crucial to recognize the human influence on coastal risk and take appropriate measures to mitigate the impacts of both climate change and human activities.

This socio-economic dependence of the risk links our results with those obtained by Brenner et al.^54^, who analysed socio-economic and environmental values in counties along the Catalan coast to define homogeneous management units to be used for integrated coastal zone management (ICZM). The homogeneous areas in their study have a similar spatial distribution to those defined here in terms of cumulative compound risk, and consequently, this will serve to introduce risk management-oriented criteria in the ICZM plans in addition to those based on the socioeconomic and environmental values obtained. In this regard, the Protocol for ICZM in the Mediterranean^55^ calls on the parties (countries) to develop natural risk prevention policies and, in particular, asks countries to undertake vulnerability and hazard assessments of coastal zones. The methods presented herein provide an easy-to-use and validated method to assess the importance of coastal risks associated with hydrometeorological and marine hazards which are especially relevant in coastal areas where the contribution of the hydrometeorological hazards to the risk in the terrestrial domain would be significant, such as the Mediterranean coast.

Finally, it has to be mentioned that the assessment of the compound coastal risk will be valid as long as the characteristics of the system (hazards, vulnerability and exposure) remain reasonably constant. Thus, the analysis should be updated when any change in the governing conditions is detected or predicted, or to integrate new observations of the variables considered.

## Methods

### Marine-induced risks

#### Hazards

The MHI index adopted to characterise the contribution of marine hazards to risks in the coastal zone follows the typical form of the CVI^56^ that has been widely used in the literature^30,46,47,57–60^. In this study, we considered the main marine hazards acting at three time-scales in the study area: episodic (storms), medium-term (decadal-scale rate of shoreline change), and long-term (sea-level rise). The index is given as MHI = (I_1_ I_2_ … I_5_/5)^½^, and the contributing components I_i_ are described below in terms of the variable used and the scale adopted.

Once the index was calculated, the resulting values were classified into five classes characterising the qualitative magnitude of the hazard and rescaled on a 1–5 scale according to the values shown in Table 5. The minimum values that delimit each class were fixed in absolute terms according to the criterion that at least four of the five indicators have a value corresponding to the class considered, and the remaining one has the lowest value (VL). For example, this implies that any combination with a value larger than 11.2 (resulting from calculating the index with four of the five indicators with a value of 5 (VH) and the remaining one with a value of 1 (VL)) is classified as VH.

##### Storms

To characterise the potential magnitude of the storms, the maximum value of the significant wave height, *Hs*, recorded in each segment along the coast over a period of time long enough to be representative of the local wave climate was selected. The data used were obtained from the SIMAR database, which extends from 1958 to the present and has a spatial resolution of approximately 0.08° (8.9 km) along the coast^61,62^. This maximum value can also be substituted by the *Hs* associated with a given return period; however, its use allows the characterisation of extreme waves without performing an extreme analysis that could be prevented in many sites because of the unavailability of the original wave time-series.

These *Hs* values were classified into five categories to characterise their contribution to the overall marine hazard intensity. For this purpose, a specific classification of storms on the Catalan coast^63^ was used, which allows the identification of potential damage in terms of erosion and flooding associated with the impact of coastal storms. The five resulting classes, VL to VH, correspond to the five categories of storms according to the used classification^63^, from weak to extreme. The intervals of the *Hs* values for each category are listed in Table 5. It must be stressed that this scale is tailored to the local conditions of the wave climate and can be adapted to other sites using values associated with other proposed storm scales for other areas^64,65^.

##### Mid-term shoreline evolution

To characterise the medium-term erosion hazard, we selected the shoreline displacement rate as a basic indicator. This was calculated for each beach along the study area using the DSAS tool^66^ with digitised shorelines from aerial photographs from the last 20 years. Shoreline evolution rates were calculated using linear regression or end-point-rate methods^67^ (depending on the number of available shorelines) in profiles approximately 100 m apart along the beach and then averaged to obtain a beach-integrated shoreline displacement rate.

The shoreline evolution rates thus obtained were then classified into five categories using an average “representative beach width” as a benchmark. This was calculated as the average value of all beach widths of less than 100 m in the study area, thus avoiding the weight of very wide beaches typical of deltaic areas, which, owing to their length, tends to underestimate vulnerability. The scale was then set by considering the resulting beach width under a given rate of evolution over a time period representative of the medium-term scale (taken as 25 years in this case). Class VH was established for erosion rates equal to or greater than those required to completely erode the “representative beach width” in 25 years. By contrast, class VL was associated with stable or accreting beaches. The ranges obtained for each class in the study area are presented in Table 5.

##### Inundation

We used the low-elevation coastal zone (LECZ) to characterise the inundation potential of the coastal zone along the study area. This zone has been widely used to identify coastal areas vulnerable to inundation at different scales, such as coastal flooding (storm-induced) and SLR^68,69^. Although this is originally defined as the land area contiguous with the coastline up to an elevation of 10 m with respect to the mean sea level^70^, in this study, we reduced its extension up to an elevation of 5 m altitude, as done in other coastal risk studies^71^, hereinafter denominated modified LECZ (mLECZ).

This factor was indicated by the slope of the mLECZ, which was calculated for each coastal segment along the study area and then classified into five categories. The hazard categories were selected based on the slope of the mLECZ required to induce inundation of a coastal strip of a given width under a reference SLR by the year 2100. The reference SLR was taken as the regional value for the AR6 SSP2-4.5 scenario (0.65 m). The cross-shore extents of the potentially inundated areas are referred to as the regional mean beach (considering all beaches), which is 67 m. Thus, class VH was established as the slope required to inundate a strip equal to the mean beach width for the zone plus 200 m under the reference SLR. This 200 m-value corresponds to the maximum extent of the protection buffer (a normative coastal setback) in the coastal zone, as defined in the Spanish coastal act^72^. The VL class corresponds to the slope required to inundate a strip, equal to the mean beach width. The intervals obtained for each class in the study area are presented in Table 5.

##### Sea-level rise

Sea-level rise is considered one of the most important forces inducing coastal hazards on a long-term scale. For the risks considered in this study, the relevant hazards are inundation and erosion, which depend on the magnitude of the forcing itself and the specific geomorphic characteristics of the coastal zone. The forcing is indicated here by the magnitude of the relative sea-level rise (RSLR), which is composed of a regional-scale projection of sea-level rise under a given climate scenario and a local component to account for possible changes in mean sea-level changes owing to subsidence. In the calculations presented in this study, we have employed tidal gauge data sourced from the REDMAR network for Spanish ports situated within the study area, spanning from 1992 to the present^73^. It is noteworthy that these data can be substituted with SLR projections to assess the potential impact of the marine hazard component under varying climate scenarios.

The magnitude of the RSLR was classified into five categories in relative terms based on the magnitude of the expected hazard with respect to the local characteristics of the coast. For this purpose, the RSLR-induced shoreline retreat for 2100 was assessed and compared with the average beach width in the study area. This was estimated using Bruun’s rule^74^ and the average shoreface slope of the study area (0.014). The resulting five classes correspond to values that induce a given shoreline retreat compared to the mean beach width (67 m). Thus, class VH corresponds to the RSLR value inducing a shoreline retreat equal to the mean beach width, whereas the VL class is limited by RSLR values inducing a shoreline retreat lower than 25% of the mean beach width. The intervals obtained for each class in the study area are presented in Table 5.

##### Shoreface slope

As mentioned above, in addition to the SLR, information on the geomorphic characteristics of the coast in also necessary to indicate the magnitude of the long-term hazard. Thus, to indicate the long-term erosion hazard potential, we selected the slope of the shoreface owing to the high sensitivity of SLR-induced retreat computations for that variable^75–77^.

To indicate its importance, we obtained the slope of the shoreface to approximately 10 m water depth, which is representative of the long-term depth of closure in the study area and has been previously used to calculate the SLR-induced erosion in the area^76,78^. This variable was classified into five categories based on the SLR-induced shoreline retreat for a shoreface with a given slope for the year 2100 under the regional value of the AR6 SSP2-4.5 scenario (0.65 m). The resulting five classes correspond to slope values that induce a given shoreline retreat compared to the mean beach width (considering all beaches) in the study area (67 m). Thus, class VH corresponds to a slope inducing a shoreline retreat equal to the mean beach width, whereas the VL class is limited by RSLR values, inducing a shoreline retreat lower than 25% of the mean beach width. The intervals obtained for each class in the study area are presented in Table 5.

#### Vulnerability

The vulnerability index (MVI) characterizes the potential of the coastal system to be harmed by the hazards presented in the previous sub-section. It measures the coast’s capacity to cope with induced impacts and, for this purpose, we considered three main components: coastal geomorphology resistance, resilience, and adaptive capacity. They were combined in the index form MVI = (I_1_ I_2_ I_3_/3)^½^, with the contributing components I_i_ described below. Once the index was calculated, the resulting values were classified into five classes characterising the qualitative magnitude of the hazard and rescaled on a 15 scale according to the values shown in Table 5. As in the case of hazards, the minimum values that delimit each class were set in absolute terms according to the criterion that at least two of the three indicators have a value corresponding to the class considered and the remaining one has the lowest value (VL).

##### Coastal geomorphology

Coastal geomorphology was used in this study as a qualitative indicator of the intrinsic vulnerability of a coastal stretch to any dynamic forcing, as it controls the magnitude of the potential response, that is, its relative erodibility. We followed the same approach as usually implemented in CVI^79^, where a coastal stretch is classified according to its geology/geomorphology into five different classes according to its susceptibility to erosion. The selected geomorphological types and their corresponding vulnerability classes are listed in Table 5. This is the most important variable and also control whether the other variables will be relevant or not.

##### Beach width/susceptibility

For segments composed of an erodible geomorphology class (from bluff to beaches), beach width is an important variable conditioning its risk to acting hazards. This variable is usually employed as a measure of the capacity of protection provided by the beach to the hinterland against the impact of marine forces^80^, such that the wider the beach is, the more protected the hinterland will be^81–84^. Thus, this variable can be used as a measure of the “resilient” capacity of the coast to cope with marine-induced hazards. It was classified into five categories as a function of the ratio of the local beach width using the expected regionally averaged shoreline retreat over 25 years as a benchmark. Thus, class VH corresponds to a beach width narrower than 25% of the expected regional-averaged shoreline retreat, including mid-term and long-term (SLR) processes (72 m). In contrast, the VL class corresponds to beaches wider than the mean erodible width in the study area. The intervals obtained for each class in the study area are presented in Table 5.

##### Accommodation space

The last variable that characterizes the vulnerability of the coast to considered hazards is the availability of accommodation space in the hinterland, that is, free space without rigid boundaries that would permit the horizontal migration and building of beaches as a response to coastal erosion or SLR, avoiding the appearance of coastal squeeze^85^. In contrast, the lack of accommodation space will lead to the disappearance of the beach under these forcings^76,86^. Thus, the existence of accommodation space can be used as a measure of the “natural capacity of adaptation” of the system. It should be noted that accommodation space is a relative concept. This is related to the expected magnitude of the shoreline retreat (and the expected migration) at a given time horizon under a given scenario^76^. In areas subjected to low erosion rates, the space required to allow landward migration should be small and vice versa. In this study and as a reference, we used a minimum extension of a 200 m-wide fringe behind the beach. Each segment was then analysed to measure the extension of the coast with such free space inland. The five resulting classes were based on the percentage of total beach length with sufficient accommodation space (Table 5).

### Hydrometeorologically-induced risks

#### Hazards

The HHI proposed in this study characterizes the magnitude of the main hazards that play a relevant role in hydrometeorological risks at different scales along the Spanish Mediterranean coast. These are the extreme rainfall characterised by the 20-years return period of the daily rainfall (T20), number of rainy days during the summer (high tourist season), number of thunderstorm days, and maximum wind gust. These were integrated in the index in the form HHI = (I_1_ I_2_ I_3_ I_4_/4)^½^, and the contributing components I_i_ are described as follows. As in the previous cases, the HHI was classified into five classes and rescaled on a 1–5 scale according to the values shown in Table 6. Following the same approach, the minimum values that delimit each class were set in absolute terms according to the criterion that at least three of the four indicators have a value corresponding to the class considered and the remaining one has the lowest value (VL).

##### 20-year return period of daily rainfall (T20)

This variable was used to characterise the heavy rainfall episodes that are responsible for flash floods. This corresponds to the daily rainfall associated with a return period of 20 years, which is the threshold that an annual extreme exceeds with a probability of 5%, and is usually employed to characterise heavy rainfalls events that are likely to occur ^87,88^. It was obtained by applying a Gumbel distribution for each AEMET station representative of each municipality based on a rainfall series of over 40 years. This was classified into five intensity classes based on the rainfall warning thresholds defined in Meteoalerta, the national Spanish weather warning system (AEMET), and the Meteorological Service of Catalonia (SMC). The resulting classifications are presented in Table 6.

##### Number of thunderstorm days

This indicator characterizes the threat to the population and infrastructure from severe weather (hail, strong winds, tornadoes, and convective storms), which can be parameterised in terms of lightning^89^ and is measured here by the number of days on which lightning is recorded in each municipality. This was calculated by counting lightning observations projected over a 1 km × 1 km matrix resolution during the period covered by the data (see “Data” section). Using this matrix, the strike density was calculated for each pixel and then averaged at the municipal level. The criteria used to classify this indicator according to the hazard level were based on quintiles and expert criteria (see Table 6).

##### Maximum wind gust

Extreme wind is an important meteorological hazard that can threaten life and infrastructures^90^. To characterise this factor, we calculated the 97.5th percentile of the daily time series of the maximum wind gusts of the available meteorological stations along the study area. Calculated values were later classified into five classes based on the wind warning thresholds used in the Spanish weather warning system (Meteoalerta) (AEMET) and SMC. The resulting classifications are presented in Table 6.

##### Number of rainy days per year during high season

This variable indicates a hydrometeorological threat affecting the recreational use of the coast (tourism), which is the main economic sector in most Mediterranean countries. Thus, in sun-and-beach destinations, the absence of rainy days is one of the elements determining the comfortability of users^91^. The high season, wherein the coastal zone (beaches) is not, was set from April to September, based on the usual influx of tourists to the study area. The variable was measured from the time-series of rainfall in meteorological stations in the study area, and values were classified according to the classes shown in Table 6.

#### Vulnerability

The hydrometeorological vulnerability index (HVI) characterizes the potential of coastal zones to be harmed by hydrometeorological hazards. Given the characteristics of the hazards considered in the study area, where flash floods are the dominant source of damage^92^, we considered three components, all of which characterise the area’s potential to favour flooding: soil permeability, slope, and stream order. They were combined in the index form MHI = (I_1_ I_2_ I_3_/3)^½^ and rescaled from to 1–5 classes following the same criterion used for the MVI.

##### Soil permeability

Soil permeability plays an important role in flooding, especially in flow velocity, and has an inverse relationship with it; a lower permeability indicates a lower infiltration capacity and a higher probability of waterlogging^93^. This was characterised by representative soil permeability at the municipal level. For this purpose, land-use classes were measured, and the 10-year return period of the runoff coefficient (Ce_10_) defined for different land use types was applied^94^ and averaged at the municipal scale. The over 100 original uses of the SIOSE database were reclassified into 15 groups according to the land use groups defined in Llasat et al.^94^. The resulting classes on the 5-interval scale are listed in Table 6.

##### Slope

The terrain slope is usually considered a key flood generation factor^95^; therefore, it is one of the main indicators of hydraulic hazards. This was characterised by a municipal average slope which was calculated from the available DTM of the study area. The 5-class scale to classify this indicator is shown in Table 6, where Meunier’s^96^ to define torrential rivers (slope ≥ 1.5%) and torrential water streams (slope ≥ 6%) was used.

##### Stream order

Finally, we also included the stream order, which indicates the level of branching of a river system, and is an indicator of the size of the drainage basin and discharge capacity; the larger the order number, the smaller is the catchment area draining to the stream^97^. In this study, the stream orders in the basins of the study area included in the CEDEX database were compared with those in areas where flash floods usually occur. The analysis revealed that streams usually experiencing flash floods tend to be of orders three to five, with 4 being the most common order. The proposed scale used to classify this indicator is presented in Table 6.

### Exposure

To assess the consequences or damage induced by the considered hazards in the coastal zone, we characterised the existing values using an exposure index, IEX. Here we adopted the “total damage approach”, which means that no vulnerability curve is associated to any value, and therefore, it characterises the potential damage. It should be noted that this is the only component common to both domains (coastal and hydrometeorological). We considered a 500 m wide-fringe along the coast (measured landwards by the DPMT, a Spanish legal setback, usually located in the landward limit of beaches) where existing values at exposure may be damaged by the considered hazards. Then, adopting an approach inspired by that presented by Viavattene et al.^83^, five components were selected to characterise values at exposure, which are described below. They were combined as IEX = (I_1_ I_2_ … I_5_/5)^½^. It should be noted that the exposure is calculated at the municipal scale such that the same exposure value is applicable to all segments within a given municipality.

Once the index was calculated, the resulting values were classified into five classes characterising the qualitative magnitude of the values at exposure and rescaled on a 1–5 scale according to the values shown in Table 7. Following the same criteria adopted for the other indicators, the minimum values that delimit each class were set in absolute terms, according to the criterion that at least four of the five indicators have a value corresponding to the class considered, and the remaining one has the lowest value (VL).

#### Land use

This component valorises existing land use in the coastal zone which may be affected by the considered hazards. Original land use types were reclassified into five categories representing the main uses in the study area, and a value from 1 to 5 was assigned to each category to reflect their importance according to the study objectives. In this case, because the analysis was oriented towards safety-related issues, the highest values were allocated to human-related land use types (see Table 7). The component IEX_LU_ was calculated as

$$IE{X}_{LU}= \sum_{j=1}^{j=n}{S}_{j} {V}_{j},$$

where *S* is the percentage of surface occupied by a given land use type *j* within the 500 m-wide buffer of the segment, *V* is the assigned value (Table 7) for such use, and *n* is the number of uses in the buffer.

#### Transport

This component measures the relative exposure of the transport network, which is one of the most important infrastructures affected, not only because of its intrinsic value but also because of the impact its disruption can have on the economy of the area^98^. This component was estimated by identifying the type of existing transportation infrastructure within the 500 m buffer zone and associating a value with it according to its importance (Table 7), an approach already used in the study area^11^. These values can be modified to reflect the importance of the different components of the transport network where it is going to be applied.

#### GFDI

To measure the relative overall economic importance of the affected area, we use the gross household disposable income, GHDI, which is a macro-magnitude measure of the income available to the residents of a territory for consumption or savings, and it is increasingly used to characterise potential economic damage of natural hazards on affected population^99^. This component is obtained from official statistics and is provided at the municipal level; therefore, any coastal segment within a given municipality will have the same associated value. Their values are classified on a 1–5 scale according to the values shown in Table 7, which were obtained by means of the quintile method.

#### Coastal economic activity

To measure the potential impacts of hazards on the coastal-related economy, we selected the most representative sector in each municipality along the study area. As expected in the Mediterranean, the dominant activity is tourism^100^, which is represented by the number of accommodation places allocated to this activity in each coastal municipality. This variable, if combined with a typical economic indicator, such as average daily expenditure of tourists, provides an estimate of the potential economic income per municipality. In addition, a few other areas exist, such as the Ebro delta, where agriculture significantly dominates tourism, even in the coastal zone. In those locations, agriculture was considered the main economic activity and a value of 5 was directly assigned to their municipalities. This economic component was classified in the range of 1–5 using the quintile method (Table 7). As with the other components, this indicator can be modified to reflect local conditions at the application site.

#### Population

Finally, following most vulnerability assessment studies, this component accounted for the population of the study area^101–103^. This value was obtained from official statistics at the municipal level and classified on a 1–5 scale using the quintile method (Table 7).

### Cumulative compound coastal risk

To obtain the risk index, the three individual sub-indices (hazard, vulnerability, and exposure) were combined as described here. First, the product of the hazard and vulnerability values, which varies between 1 and 25, was reclassified according to the rules presented in Table 8, resulting in a nonlinear reclassification wherein the limits for each class from VL to VH are given by (≤ 2, ≤ 5, ≤ 9, ≤ 15, > 15). The resulting values, once reclassified on a scale of 1–5, were then combined with the exposure index following the same rules to obtain the risk index. This was performed individually for the marine (MRI) and hydrometeorological (HRI) domains. Finally, the cumulative compound risk index (CCRI) was obtained in the same manner by combining the calculated marine and hydrometeorological risk indices.

In addition, the cumulative compound risk was mapped using the k-means clustering technique. In the context of coastal hazard and vulnerability analysis, different implementations of this technique exist, such as applying basic data to identify classes of different profiles^104^, reducing input data prior to index development^105^, or aggregating data prior to identifying existing classes^106^. For comparison with the CCRI-based classification, we classified coastal municipalities into five groups based on three seeding variables: marine and hydrometeorological hazards and vulnerabilities (defined as the product MHI·MVI and HHI·HVI, respectively), and exposure along the coast (IEX). In all cases, these variables are used once they were rescaled to 1–5 according to the procedures described above.

The main difference with respect to index-based classification is that, instead of ranking municipalities according to their risks, mapping is based on the similarities and differences in the components contributing to the compound risk. The objective was to group coastal municipalities according to similar attributes of marine and hydrometeorological risks that could be used to define coherent management strategies.

### Index validation

The coastal vulnerability and/or risk calculated using a given index is the result of applying a given equation and an associated scale. To estimate its usefulness in representing actual risk conditions, we validated it for the study area. Because the calculated risk integrates the consequences of all hazards acting at different time scales, we performed a double validation using different types of data to characterise the damage.

First, we used the compensatory payments made by the Consorcio de Compensación de Seguros (CCS), which is an instrument in the service of the Spanish insurance sector that, among other functions, covers extraordinary risks, such as extraordinary floods, coastal storms (sea battering), and atypical cyclones^107^. The last one includes the action of violent tropical cyclones, intense cold lows, tornadoes and extreme winds^108^. Thus, we compared the compensatory payments by CCS associated with these natural hazards aggregated at the province scale, with the province-aggregated value of the cumulative compound risk index. This value was calculated by adding all CCRI values of the coastal municipalities composing each province. Insurance data have been successfully used as proxies for economic damage from floods^109,110^.

In addition, a second qualitative validation exercise was performed by comparing the province-aggregated CCRI with qualitative data on the damage associated with hydrometeorological and marine hazards in the coastal municipalities of the study area. The approach was to assess the cumulative observed/reported damages and rank them on a qualitative scale of 1 to 5, reflecting their importance. This was assessed separately for the marine and hydrometeorological domains and then added to provide a qualitative measure of their importance. Finally, the cumulative compound damage was obtained by adding hydrometeorological and marine-induced damage, the damage index, and reclassifying them on a 1–5 scale. In the case of hydrometeorological damage, we counted the number of damaging floods in each municipality incorporated in the Inungama database^42^. The range of the number of floods was then divided into five classes using equal-space intervals. For this purpose, the number of floods in the municipality of Barcelona was extracted to avoid overweighting, as the number of floods recorded was approximately 1.8 times that of the maximum value for the other municipalities. The lowest class was formed by the number of municipalities without floods. For marine hazards, we used two sources of qualitative data: intensity of damage reported by technical personnel of coastal municipalities of Catalonia to be representative of current conditions actual conditions^43^; and damage registered during the impact of the extreme coastal storm Gloria^18,19^. The occurrence of different types of damage in the coastal zone associated with these hazards was identified and scored according to the criteria listed in Table S5 (Supplementary Information). The overall marine damage was determined by averaging the resulting values for each type of data.

### Supplementary Information

Supplementary Information.

## Data Availability

All data used to inform this study are available from open-source databases identified in Table 2. All data generated from this study are available from the corresponding author on reasonable request.
