# Supplementary material for: Mapping cumulative compound hydrometeorological and marine-induced risks on the NW Mediterranean coast
Source: Sci Rep. 2024 Feb 8;14:3237. doi: 10.1038/s41598-024-53899-z (PMC10853219; doi:10.1038/s41598-024-53899-z)
Supplement: Supplementary file 1 — Supplementary Information. [file 41598_2024_53899_MOESM1_ESM.pdf]

Supplementary Information for

Mapping cumulative compound hydrometeorological and marine induced risks in the NW Mediterranean coast

Rut Romero-Martín[1], Isabel Caballero-Leiva[2], M. Carmen Llasat[2], Montserrat Llasat[2], Tomeu Rigo[3], Herminia I Valdemoro[1], Joan Gilabert[4], María Cortès[5], José A Jiménez[1*]

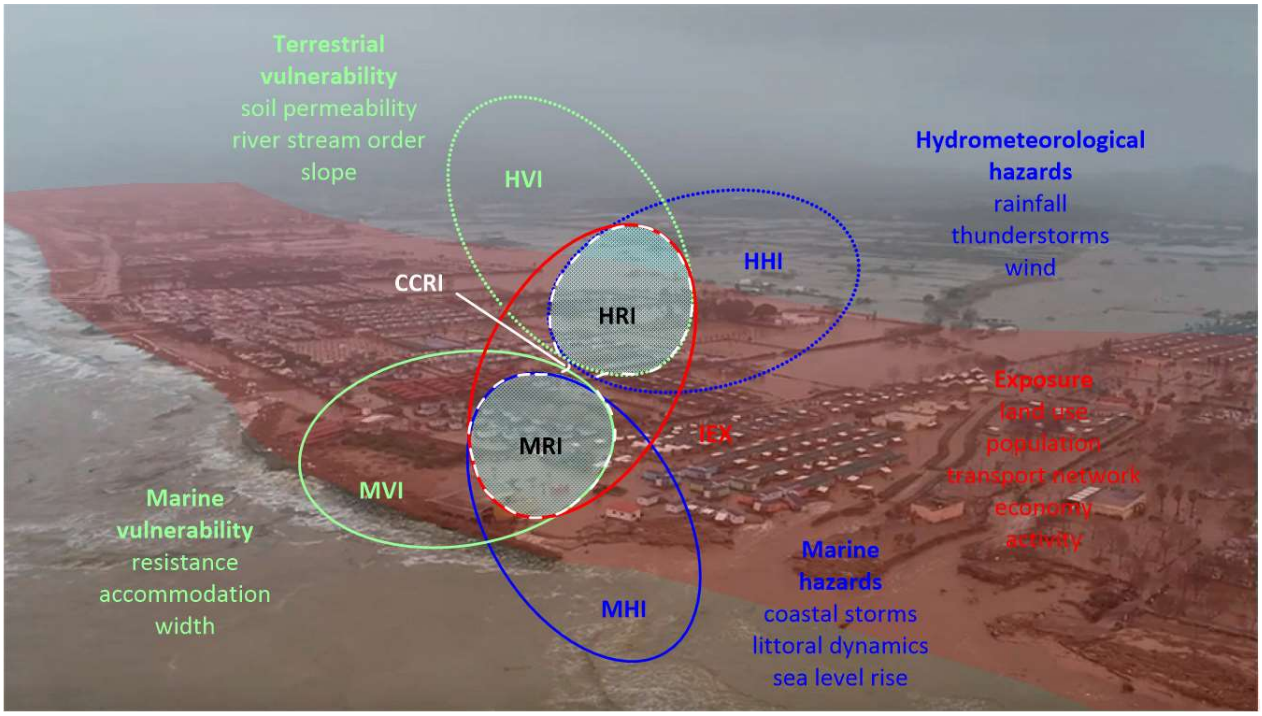

Figure S1. Conceptual framework of the cumulative compound risk index (CCRI). The marine component is given by the Marine Hazard Index (MHI) and Marine Vulnerability Index (MVI) which determine the Marine Risk Index (MRI). The hydrometeorological (terrestrial) component is given by the Hydrometeorological Hazard Index (HHI) and Hydrometeorological Vulnerability Index (HVI) which determine the Hydrometeorological Risk Index (HRI). They act over a common exposed area, which is a 500 m-wide buffer along the coast (represented by the red shadowed area). The level of exposure of this area is characterized by the Exposure Index (IEX). The background photo shows the compound flooding of the Tordera delta (Barcelona) during the impact of the storm Gloria in January 2020 (background photo: Agents rurals, Generalitat de Catalunya). The figure was created with Grapher 16.9.3 software (www.goldensoftware.com)

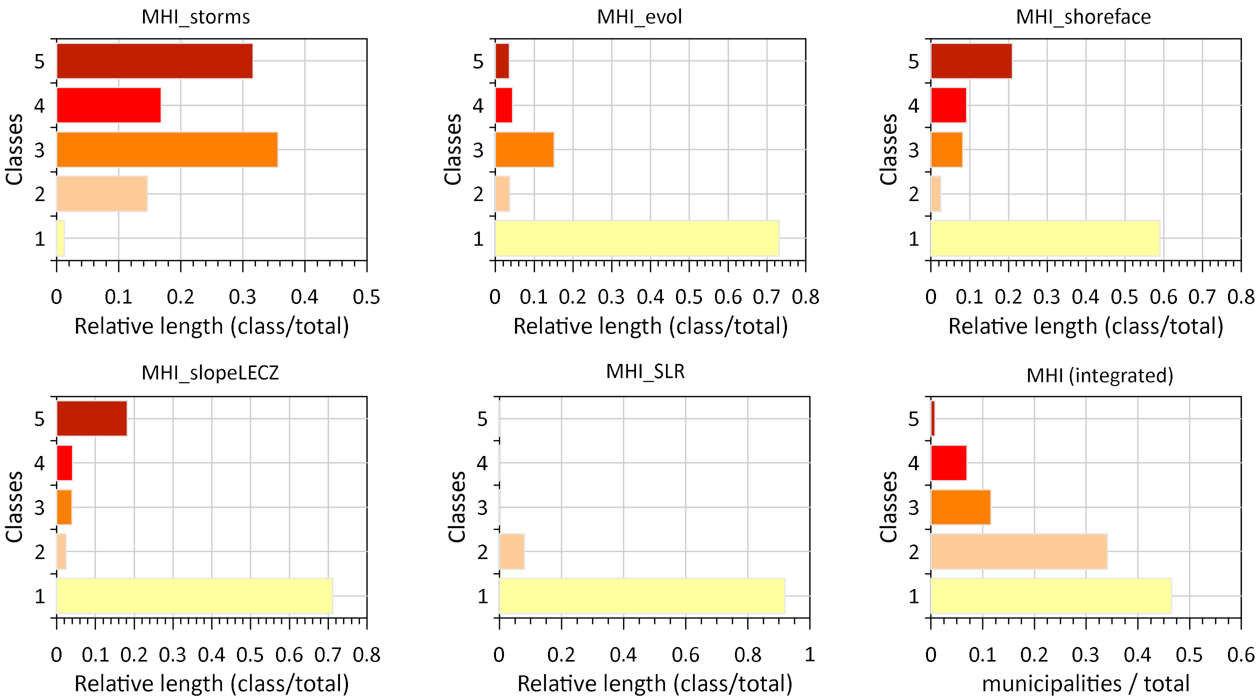

Figure S2. Fraction of the shoreline length of the study area belonging to each hazard class (1: VL, 2: L; 3: M; 4: H; 5: VH) for marine hazard indicators (storms; mid-term shoreline evolution; inundation / slope of the LECZ; SLR; shoreface slope); and for integrated hazard index (MHI) expressed in fraction of municipalities.

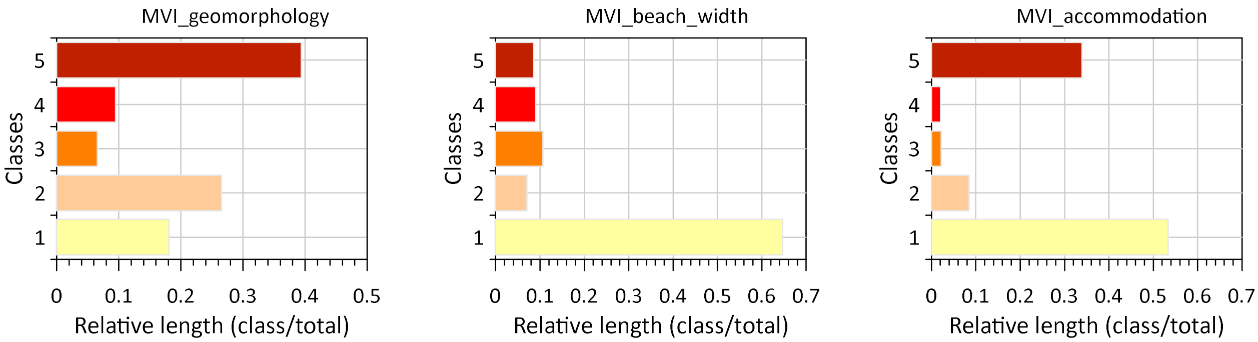

Figure S3. Fraction of the shoreline length of the study area belonging to each vulnerability class (1: VL, 2: L; 3: M; 4: H; 5: VH) for marine vulnerability indicators (coastal geomorphology; beach width / susceptibility; accommodation space).

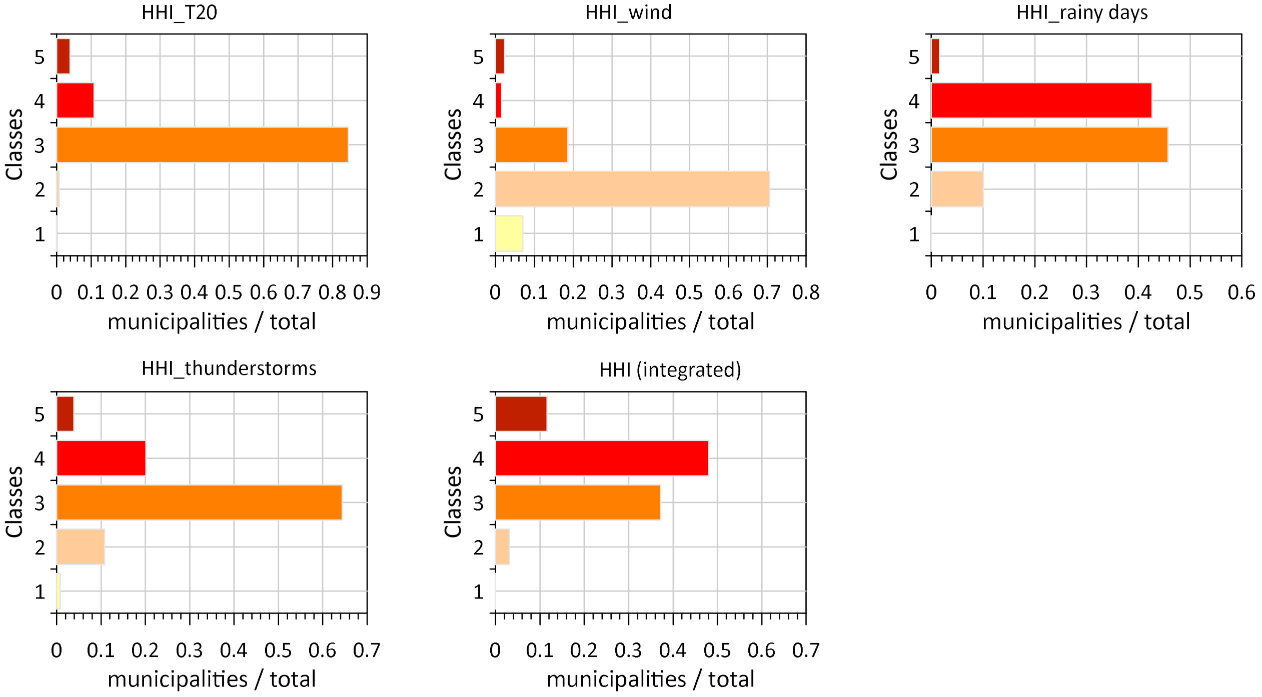

Figure S4. Fraction of municipalities of the study area belonging to each hazard class (1: VL, 2: L; 3: M; 4: H; 5: VH) for hydrometeorological hazard indicators (20-year Tr of daily rainfall; thunderstorm days; maximum wind gust; rainy days during high season) and integrated hazard index (HHI).

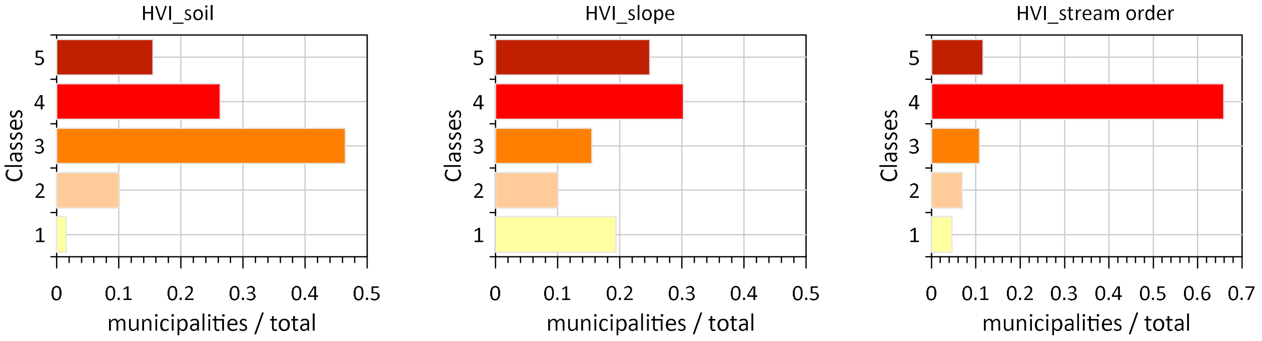

Figure S5. Fraction of municipalities of the study area belonging to each vulnerability class (1: VL, 2: L; 3: M; 4: H; 5: VH) for hydrometeorological vulnerability indicators (soil permeability; slope; stream order).

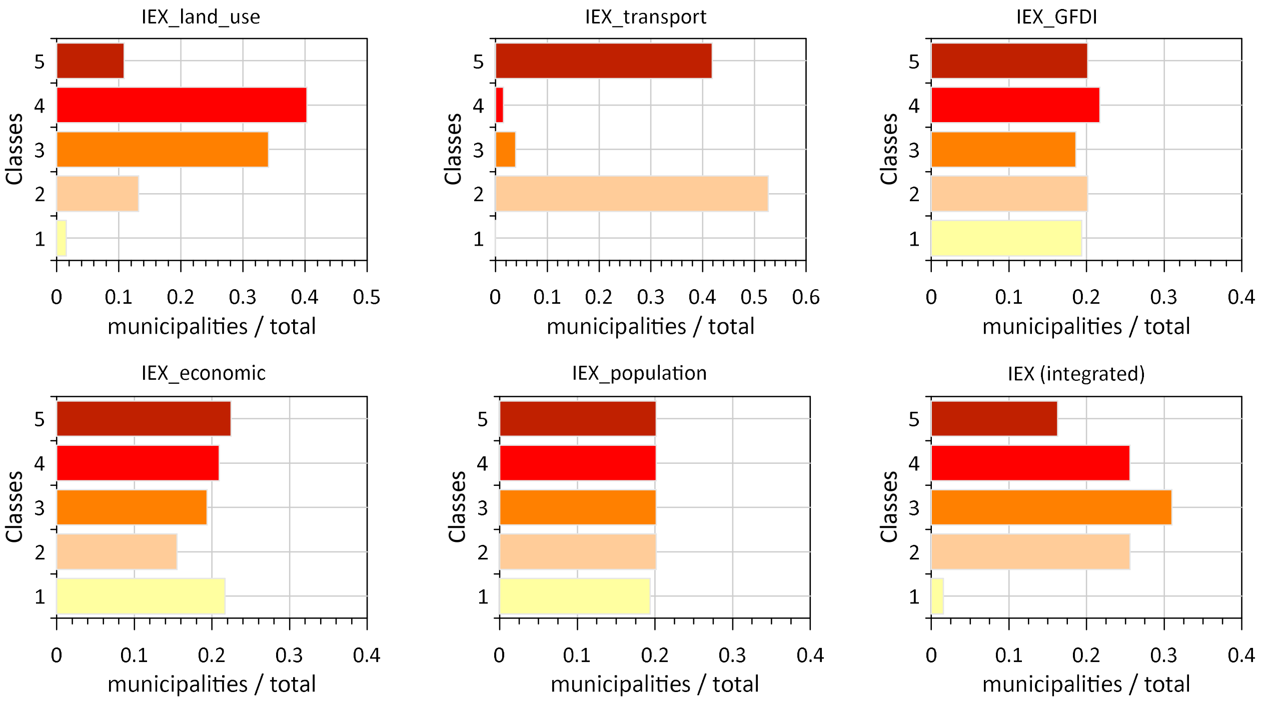

Figure S6. Fraction of municipalities of the study area belonging to each exposure class (1: VL, 2: L; 3: M; 4: H; 5: VH) (land use; transport network; GFDI; economic activity; population) and integrated exposure (IEX). The indices GFDI, economic and population have been classified according to the quintile method.

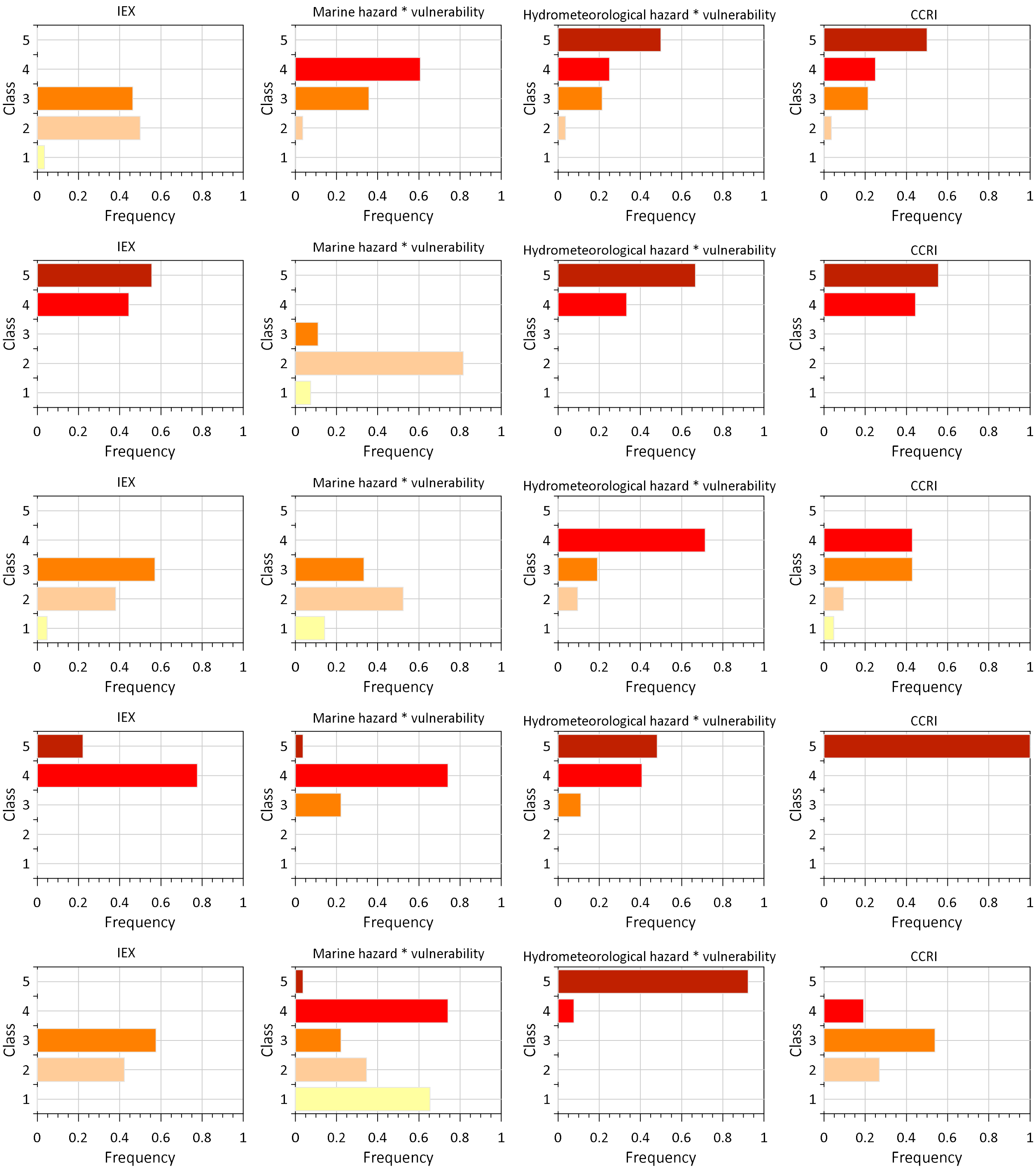

Figure S7. Fraction of municipalities of each cluster belonging to each exposure, and marine and hydrometeorological hazard * vulnerability classes; cumulative compound coastal risk -CCRI- (1: VL, 2: L; 3: M; 4: H; 5: VH). From top to bottom, clusters/number of municipalities (1/28; 2/27; 3/21; 4/27; 5/26).

Table S1. Number of municipalities for all range of marine hazards *vs* vulnerabilities in the study area.

		Marine Hazard Index					Total
		VL	L	M	H	VH	
Marine Vulnerability Index	VL	13	0	0	0	0	13
	L	9	4	0	0	0	13
	M	16	5	5	0	1	27
	H	11	7	4	1	0	23
	VH	11	28	6	8	0	53
	Total	60	44	15	9	1	129

Table S2. Number of municipalities for all range of hydrometeorological hazards vs vulnerabilities in the study area.

		Hydrometeorological Hazard Index					Total
		VL	L	M	H	VH	
Hydrometeorological Vulnerability Index	VL	0	0	2	0	0	2
	L	0	0	3	2	0	5
	M	0	0	7	5	1	13
	H	0	1	3	3	0	7
	VH	0	3	33	52	14	102
	Total	0	4	48	62	15	129

Table S3. Number of municipalities for all range of marine and hydrometeorological risks in the study area.

		Hydrometeorological Risk Index					Total
		VL	L	M	H	VH	
Marine Risk Index	VL	0	1	2	7	0	10
	L	0	1	4	14	2	21
	M	0	1	10	17	10	38
	H	0	0	4	15	20	39
	VH	0	0	0	2	19	21
	Total	0	3	20	55	51	129

Table S4. Main risk characteristics per province along the study area. (CCRI: cumulative compound risk, MRI: marine risk; HRI: hydrometeorological risk; IEX: exposure; MHV: marine hazard·vulnerability; HHV: hydrometeorological hazard· vulnerability; clusters: number of municipalities per cluster C1/C2/C3/C4/C5; % coastline with C2 & C4: fraction of coastline of the province with the riskiest clusters C2 and C4).

Province	Municipalities	CCRI	MRI	HRI	IEX	MHV	HHV	clusters	% coastline with C2 & C4
Girona	22	3.4	2.3	4.1	2.9	1.7	4.9	1/2/2/2/15	0.16
Barcelona	27	4.9	4.3	4.8	4.2	3.1	4.5	4/10/0/12/1	0.93
Tarragona	21	4.4	3.7	4.5	3.5	3.0	4.7	676/1/5/3	0.36
Castelló	16	3.6	3.2	3.8	2.8	3.3	4.4	8/3/1/2/2	0.39
Valencia	24	4.0	3.3	3.7	2.9	3.4	3.8	8/2/9/5/0	0.54
Alicante	19	3.7	2.8	4.1	3.2	1.9	4.2	1/4/8/1/5	0.39

Table S5. Scale for marine damage index for qualitative validation of indexes

Damages in the coastal zone due to marine hazards were catalogued in base of a survey performed to technical personnel of all coastal municipalities of Catalonia (CIIRC, 2010). The following table show the possible answers to the identification of relevant damage associated with marine hazards in their municipalities during the last years.

Table S5a. Possible damage types and score

Type	Damage	Score
a	Nothing	0
b	Shoreline fluctuation	0
c	Significant beach erosion	3
d	Beach furniture	4
e	Promenade/waterfront/infrastructures	5

Table S5b.Possible combinations, score and damage classes

Type	Score	Class
e+d+c	12	5
e+d	9	4
e+c	8	
d+c	7	3
3	5	
d	4	
c	3	2
a / b	1	1
